# Ionomer-Modulated Electrochemical Interface Leading to Improved Selectivity and Stability of Cu_2_O‑Derived Catalysts for CO_2_ Electroreduction

**DOI:** 10.1021/acscatal.5c01614

**Published:** 2025-05-23

**Authors:** Matt L.J. Peerlings, Maaike E.T. Vink-van Ittersum, Jan Willem de Rijk, Petra E. de Jongh, Peter Ngene

**Affiliations:** Materials Chemistry and Catalysis, Debye Institute for Nanomaterials Science, 8125 Utrecht University, 3584 CG Utrecht, The Netherlands

**Keywords:** electrochemical CO_2_ reduction, Cu catalyst, ionomer, catalyst microenvironment, stability, electrochemical surface modification

## Abstract

Copper is an attractive catalyst for the electrochemical reduction of CO_2_ to high value C_2+_ products such as ethylene and ethanol. However, the activity, selectivity and stability of Cu-based catalysts must be improved for industrial applications. In this work, we investigate the effects of ionomers on the microenvironment and consequently the catalytic performance of Cu_2_O particles with a well-defined cubic shape. Cu_2_O particles without an ionomer coating were compared to those with a Nafion-based cation-exchange layer (CEL) and a Sustainion-based anion-exchange layer (AEL), as well as electrodes with two successive layers of Nafion and Sustainion in either order. Using these model electrodes, we found that the selectivity to C_2+_ products is significantly improved with a Nafion coating, regardless of whether it is in direct contact with the copper surface or present as an overlayer on top of chloride-exchanged Sustainion. The selectivity improvement by Nafion is ascribed to the exclusion of proton-donating bicarbonate ions, which limits the competing hydrogen evolution reaction. Interestingly, introducing a second layer of Sustainion causes a selectivity shift from ethylene to ethanol. In addition, improved catalyst stability is observed for the Nafion-containing electrodes due to a mitigation of potassium bicarbonate precipitation and copper agglomeration. These results demonstrate that regulating the catalyst microenvironment via ionomer coatings is a promising approach to designing electrodes with superior and tunable catalytic performance.

## Graphical abstract

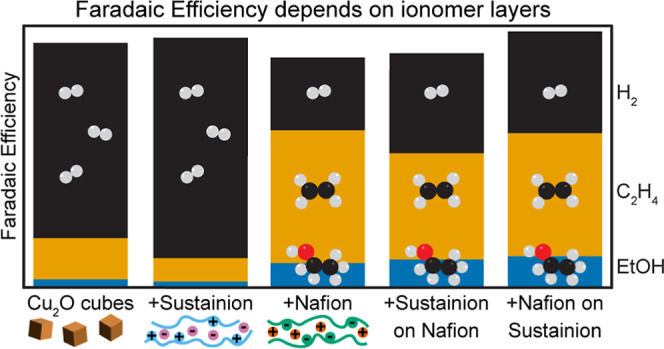

## Introduction

Excessive global CO_2_ emissions have created a great need for CO_2_ capture and utilization technologies that can aid in closing the carbon cycle. An interesting CO_2_ utilization approach is using renewable electricity to electrochemically reduce CO_2_ (CO_2_RR) into useful products.^1^ Although several catalysts are active for this reaction, only copper-based catalysts can make value-added C_2+_ products like ethylene and ethanol in significant amounts.^2,3^ However, the selectivity of Cu-based catalysts to C_2+_ products is not yet sufficient for industrial application, mainly due to the competing hydrogen evolution reaction (HER).^1,4^ Additionally, catalyst stability is an important issue which must be addressed.^5^

Different strategies have been pursued to improve the catalytic performance of copper-based CO_2_RR electrocatalysts, including the use of oxide-derived Cu,^1,6–8^ alloying/mixing with other metals or oxides^9,10^ and tuning the copper morphology in terms of exposed facets^11,12^ and higher surface roughness.^13^ Besides the catalyst, the electrolyte composition and specifically, the local microenvironment at the catalyst/electrolyte interface strongly affects the catalytic performance.^14^ For example, cations play an essential role in enabling the CO_2_RR by electrochemically stabilizing negatively charged reaction intermediates on the copper surface.^15^ However, at low bicarbonate buffer concentrations (≤0.1 M KHCO_3_) they also enable the competing water-mediated HER, whereas their effect on the bicarbonate-mediated HER at high buffer concentrations is limited.^16^ Unfortunately, studying catalyst and microenvironment effects is complicated because these effects are often intertwined. For example, a higher surface roughness generally leads to a higher CO_2_RR activity but this also generates a higher local pH and cation concentration near the catalyst surface.^17^

The importance of controlling the catalyst’s microenvironment has been increasingly recognized in recent research.^18,19^ A common strategy is to apply ion-selective ionomer coatings. Using anion-selective imidazolium-based ionomer coatings on an Ag-based catalyst, Koshy et al.^20^ found that the competing HER was promoted, while the CO_2_RR to CO was unaffected. They ascribed this undesired result to the anion exchange layer (AEL) improving local availability of HCO_3_^–^ ions, which promote proton donation and reduction to H_2_. On the other hand, applying a cation-exchange layer (CEL) composed of the ionomer Nafion improves the CO_2_RR selectivity to C_2+_ products, although explanations for this phenomenon vary.^21–23^ For instance, by varying the ink solvent used to deposit the catalyst particles on an electrode substrate, de Sousa et al.^21^ showed that a higher C_2+_ product selectivity is obtained when only part of the copper particles are covered in Nafion, and part is still exposed to the electrolyte. Therefore, they concluded that the acidity and effective proton transport by Nafion lower the C_2+_ product selectivity. In contrast, Ding et al.^22^ argue that a thicker CEL suppresses Cu restructuring and optimizes local CO_2_ and H_2_O concentrations, leading to more selective and stable catalytic performance. Kim et al.^23^ also reported a selectivity-promoting effect of Nafion, but ascribed this to a higher local pH during electrolysis, mediated by electrostatic repulsion of the negatively charged sulfonate functional groups of the Nafion trapping hydroxide ions at the electrochemical interface. Despite these divergent explanations, it is clear that Nafion impacts the catalyst’s selectivity to C_2+_ products.

Interestingly, Kim et al.^23^ showed that combining CELs and AELs can yield even further improvement of the catalytic performance. In contrast to the findings of Koshy et al. for an Ag-based system,^20^ they found that the addition of a Sustainion-based AEL improves the CO_2_RR activity. They ascribed this to an improvement in the local CO_2_/H_2_O ratio because Sustainion has a higher CO_2_ solubility than water. Notably, they demonstrated that using a bilayer of CELs and AELs, the C_2+_ product selectivity could be improved even further. This was ascribed to the synergistic effects of combining both layers, featuring a high CO_2_/H_2_O ratio and high local pH.^23,24^

Such a bilayer system appears analogous to a bipolar membrane-based (BPM) system. Petrov et al.^25^ discuss that in the CEL-AEL or reverse-bias configuration, water dissociates at the CEL/AEL boundary into H^+^ and OH^–^, which travel to the cathode and anode, respectively. Besides an inner CEL blocking (bi)­carbonate ions, the resulting more acidic microenvironment around the copper cathode liberates CO_2_ from bicarbonate ions. This limits undesired (bi)­carbonate precipitation, but also lowers the catalyst selectivity. Intriguingly, such a selectivity decrease was not observed by Kim et al.^23^ for the CEL-AEL electrode. Petrov et al.^25^ discuss that in the AEL-CEL or forward-bias configuration, hydroxide ions travel from the cathode and protons travel to the anode, reacting to form water at the AEL/CEL boundary. The advantage is a more basic microenvironment that benefits the catalyst selectivity, but stability issues arise from (bi)­carbonate precipitation in the AEL, and delamination and blistering at high current densities because of water being generated at the AEL/CEL exchange layer.

Based on these previous works, it is clear that ionomer layers can strongly affect the catalytic performance of Cu-based electrodes. However, their effects on the catalyst selectivity and stability are not yet fully understood, especially the bilayer systems. In this work, we further study the application of multiple ionomer layers on the catalytic performance of Cu-based electrodes. We show that the selectivity to C_2+_ products is governed by the presence or absence of a Nafion layer, whereas introducing an additional Sustainion layer increases the ethanol to ethylene ratio. We rationalize these findings based on the selective transport of ions during CO_2_ electrolysis mediated by the ionomer layers. Additionally, marked differences in catalyst stability are observed and discussed based on catalyst detachment, restructuring and formation of bicarbonate deposits. Our results show that applying ionomer coatings is an effective strategy for controlling the catalyst microenvironment and hence the electrocatalytic CO_2_RR performance.

## Experimental Section

### Chemicals

Copper­(II) chloride dihydrate (CuCl_2_·2H_2_O, 99.0%), sodium hydroxide (NaOH, 97%), hydroxylamine hydrochloride (NH_2_OH·HCl, 99.995%), isopropanol (99.5%), Nafion D-520 dispersion (5 wt %, ≥1.00 mequiv/g exchange capacity), nitric acid (HNO_3_, 70%) and potassium bicarbonate (KHCO_3_, ≥99%) were purchased from Sigma-Aldrich, sodium dodecyl sulfate (SDS, 99%) was bought from Acros Organics and Sustainion XA-9 dispersion (5 wt %) was acquired from dioxide materials.

### Synthesis of Cu_2_O Cubes

Cube-shaped Cu_2_O nanoparticles with {100} faces were prepared using a colloidal synthesis method based on the procedure of Huang et al.^26^ Typically, 89 mL Milli-Q was added to a 250 mL round-bottomed flask and kept at 33 °C using a water bath. Subsequently, 5.0 mL of a 0.1 M CuCl_2_ solution and 0.87 g SDS were added under magnetic stirring. Once all SDS had dissolved, 1.8 mL of a 1.0 M NaOH solution was added, turning the solution light blue because of the formation of Cu­(OH)_2_ precipitate. Lastly, 4.0 mL 0.1 M NH_2_OH·HCl solution was added to the mixture. After 1 min of stirring to ensure homogeneous mixing, the magnetic stirring was stopped and the solution was aged for 1 h. During this growth period, the solution turned from blue to green to orange, indicating reduction of the Cu^2+^ species and formation of the desired Cu_2_O nanocrystals. The particles were washed three times in a 50:50 Milli-Q/ethanol mixture by centrifuging at 4500 rpm and decanting, followed by a similar washing step in only ethanol. The particles were stored dispersed in ethanol.

### Preparation of Electrodes

Glassy carbon electrodes (SIGRADUR K discs, HTW Hochtemperatur-Werkstoffe GmbH) were cleaned by storing them overnight in a 5% HNO_3_ solution. After thorough washing with Milli-Q water, the electrodes were mechanically polished using a diamond polish with decreasing particle size, starting with 1 μm followed by 0.25 and 0.05 μm (MetaDi Supreme, Buehler). Lastly, the electrodes were sonicated for 15 min in Milli-Q water to remove any polish residue.

Electrodes without ionomer were prepared by drop-casting the Cu_2_O cubes in ethanol dispersion on a glassy carbon to achieve a 0.1 mg/cm^2^ loading. All other electrodes were prepared by first drying the Cu_2_O cubes to allow for weighing the desired amount of catalyst particles. Catalyst inks were prepared by mixing 4 mg of dried Cu_2_O catalyst with 3.2 mL Milli-Q, 600 μL isopropanol and 100 μL Nafion (Naf) or Sustainion (Sus) dispersion. The inks were sonicated for 15 min, after which 700 μL was drop-cast onto a glassy carbon electrode to achieve a 0.1 mg/cm^2^ Cu_2_O catalyst loading and 54 wt % binder with respect to the Cu_2_O catalyst. Doubly layered Naf-Sus and Sus-Naf electrodes were prepared following the same procedure for the second layer, but with the other ionomer (Sustainion and Nafion, respectively) and without Cu_2_O catalyst in the ink. Thus, the total ionomer loading of the doubly layered Naf-Sus and Sus-Naf electrodes was 70 wt % with respect to the Cu_2_O catalyst.

Before use, the Sustainion and Naf-Sus electrodes were immersed for 1 h in 0.1 M KHCO_3_ to exchange Cl^–^ for HCO_3_^–^ counterions and washed thoroughly with Milli-Q water.

### Structural Characterization

X-ray Diffraction (XRD) measurements were performed on a Bruker D2 Phaser equipped with a Co K_α_ X-ray source (λ = 1.79026 Å). Low-magnification SEM–EDX images and maps were taken on a Zeiss EVO 15 operated at 20 kV and 500 pA, equipped with secondary electron detector. High-magnification SEM–EDX images and maps were made on a FEI Helios G3 UC microscope operated at 5 kV in immersion mode.

### Electrocatalytic Experiments

All electrocatalytic experiments were carried out in a custom-build electrochemical H-cell (Figure S1). Both the anode and cathode compartments were filled with 17 mL 0.1 M KHCO_3_ electrolyte, leaving 1 mL headspace on either side. The electrolyte was stored prior to the measurements in the presence of Chelex (100 sodium form, Sigma-Aldrich) to remove any metal impurities. Furthermore, the electrochemical cell was stored in 5% HNO_3_ and thoroughly cleaned with Milli-Q water in between measurements to avoid metal contaminations. The cathodic and anodic compartments were flushed at least 30 min before and during all electrocatalytic experiments with 20 mL/min CO_2_ and Ar, respectively. Both compartments were separated by a Fumasep FAA-3-PK-130 (Fumatech BWT GmbH) anion exchange membrane (AEM). A three-electrode configuration was used in combination with a Parstat potentiostat, using a commercial IrO_2_-based anode electrode (dioxide materials) and a Ag/AgCl reference electrode (Metrohm) located near the working electrode. Both the working and counter electrode had 3.8 cm^2^ geometric surface area exposed to the electrolyte. All electrode potentials were converted to the RHE scale and *iR* corrected according to the following formula

$$E_{RHE}=E_{Ag/AgCl}+0.210+0.059\times pH-iR$$

Electrochemical impedance spectra were taken before and after electrolysis to obtain the average Ohmic resistance used for *iR* correction and to verify that the resistance stayed constant throughout the experiment. Chronoamperometry measurements were performed to determine the catalyst performance while applying about 85% *iR* compensation during each measurement, with the remainder corrected afterward.

### Product Quantification

Gaseous products were analyzed using an online gas chromatograph (Global Analysis Solutions Microcompact GC 4.0), which was equipped with three channels. Channel 1 was equipped with an Rt-QBond (10 m*0.32 mm, Agilent) packed column and an FID detector for the detection of small hydrocarbon molecules such as CH_4_, C_2_H_4_ and C_2_H_6_. Channel 2 was equipped with a Molecular Sieve 5 A (10 m* 0.53 mm, Restek) packed column and an FID detector with a methanizer to increase the detection sensitivity of CO. Channel 3 was equipped with a Carboxen 1010 (8 m*0.32 mm, Agilent) packed column and TCD detector for H_2_ detection. High purity nitrogen (N_2_; 99.999%) was used as carrier gas. The obtained peak areas were converted to Faradaic efficiency (FE) values using the following formula.

$$FE\left(\%\right)=\frac{C_{x}\times q\times n_{e}\times F\times 10^{-9}}{V_{M}\times i_{tot}}\times 100\%$$

In which *C*_*x*_ is the volumetric concentration of product *x* in ppm as determined from the peak area and calibration curve of the GC, *q* is the gas flow rate in mL min^–1^, *n*_*e*_ is the number of electrons transferred for each product, *F* is the Faraday constant (96,485C mol^–1^), *V*_M_ is the molar volume of an ideal gas at the given conditions (22.4 L mol^–1^), *i*_tot_ is the measured total current in A and 10^–9^ is a correction factor to convert to standard units.

Products remaining in the liquid phase were analyzed using a 400 MHz VNMRS-400 Varian NMR. Samples tubes were filled with a mixture of 500 μL electrolyte and 100 μL internal standard solution containing 10 mM DMSO and 50 mM phenol in D_2_O. By comparing the product and internal standard signals, the product concentrations inside the NMR tubes and inside the catholyte were obtained. These concentrations were converted to Faradaic efficiency according to the formula

$$FE\left(\%\right)=\frac{C_{x}\times n_{e}\times {F\times V}_{catholyte}}{i_{tot}\times t}$$

With *C*_*x*_ the concentration of product *x* in mol L^–1^ inside the electrochemical cell, *V*_catholyte_ the catholyte volume in *L* and *t* the time in s to form product *x*. A more detailed calculation can be found in the Supporting Information.

### Determination of Electrochemical Surface Area (ECSA)

The electrochemical surface area (ECSA) was determined during the catalytic measurements by performing double layer capacitance (DLC) measurements after fixed electrolysis time intervals of 0, 1, 3, 6, and 20 h. The DLC measurements were carried out following the procedure described by Morales and Risch^27^ and Vos et al.^28^ Specifically, the measurements were performed between −0.2 and +0.3 V vs RHE at scan rates of 200 −1400 mV/s. The capacitance values were determined via the slope obtained from an allometric fit of the current width between the anodic and cathodic scans as a function of scan rate. To obtain the ECSA, the capacitance was divided by that of a reference Cu foil (6.0 μF/cm^2^).

## Results and Discussion

### Structural Characterization of Cu_2_O Cubes

Well-defined cubic-shaped Cu_2_O particles were used for this study as they offer a good opportunity to investigate structural evolution under CO_2_RR conditions. The particles were prepared via the colloidal synthesis procedure first reported by Huang et al.^26^ Subsequently, the particles were deposited on a glassy carbon substrate as an ink which contains the Cu_2_O particles and either Nafion or Sustainion as a binder in a Milli-Q/isopropanol solvent mixture (84 vol % Milli-Q). To verify whether the synthesis was successful, the as-prepared cubes were characterized using XRD, shown in Figure 1. In this figure, it is clear that the (200) peaks are more intense than the (111) and (220) peaks, especially upon comparison to the Cu_2_O PDF.^29^ This confirms that the Cu_2_O particles expose mainly {100} faces and therefore have a cubic shape. The (200) peak is also preferentially exposed for the Cu_2_O cubes deposited on a glassy carbon substrate, regardless of the type of ionomer used.

**Figure 1:**
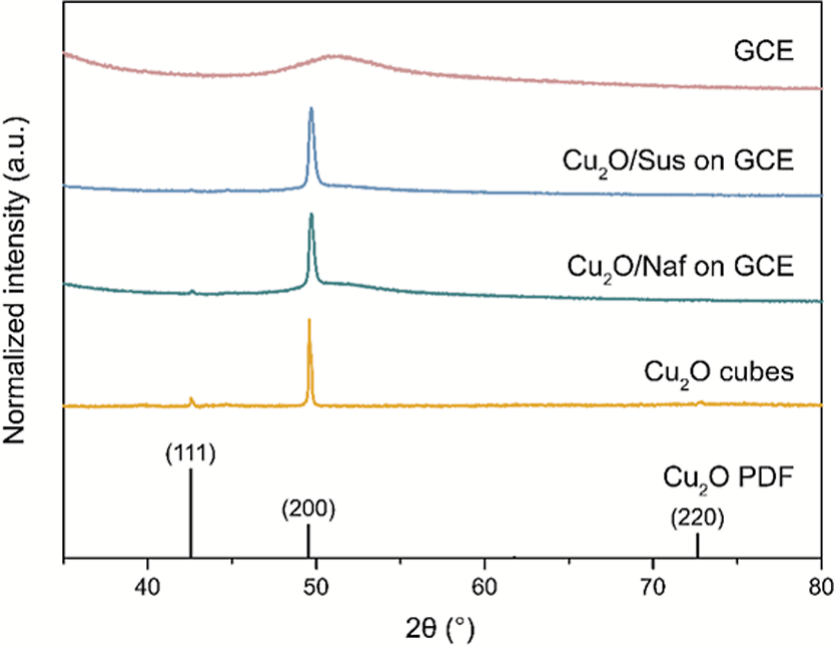
XRD patterns of as-prepared Cu_2_O cubes compared to a Cu_2_O reference, showing a clear preferential orientation in the (200) direction. The Cu_2_O cube-based electrode does not show clear Cu_2_O-related peaks because of interference from the glassy carbon electrode (GCE) background signal.

To further characterize the Cu_2_O cubes and electrodes, SEM images were acquired, as shown in Figures 2 and S3–S6. Figure 2A shows the as-synthesized Cu_2_O particles, confirming their cubic shape. The particle size distribution was quantified by assessing the edge length of individual cubes. As given in Figure S2, an average size of 581 ± 71 nm was obtained. Figure 2B shows the as-prepared electrodes with Nafion binder. Bright spots are observed for the Cu_2_O cubes, although the contrast is much lower than in Figure 2A. The reason for this is that the particles are fully covered by the Nafion binder, obscuring the signal. By assuming a wetted Nafion film thickness of 1.58 g/cm^3^,^30^ the intended Nafion layer thickness would correspond to 750 nm, which is larger than the size of the Cu_2_O cubes. The EDX map in Figure 2C confirms the presence of copper related to the Cu_2_O cubes, and F related to the PTFE backbone of the Nafion binder. Similarly, the Sustainion binder covers the Cu_2_O cubes as shown in Figure S4. However, the EDX signal of N is very weak due to few nitrogen atoms being present in Sustainion, in contrast to the abundance of F atoms in Nafion.

**Figure 2:**
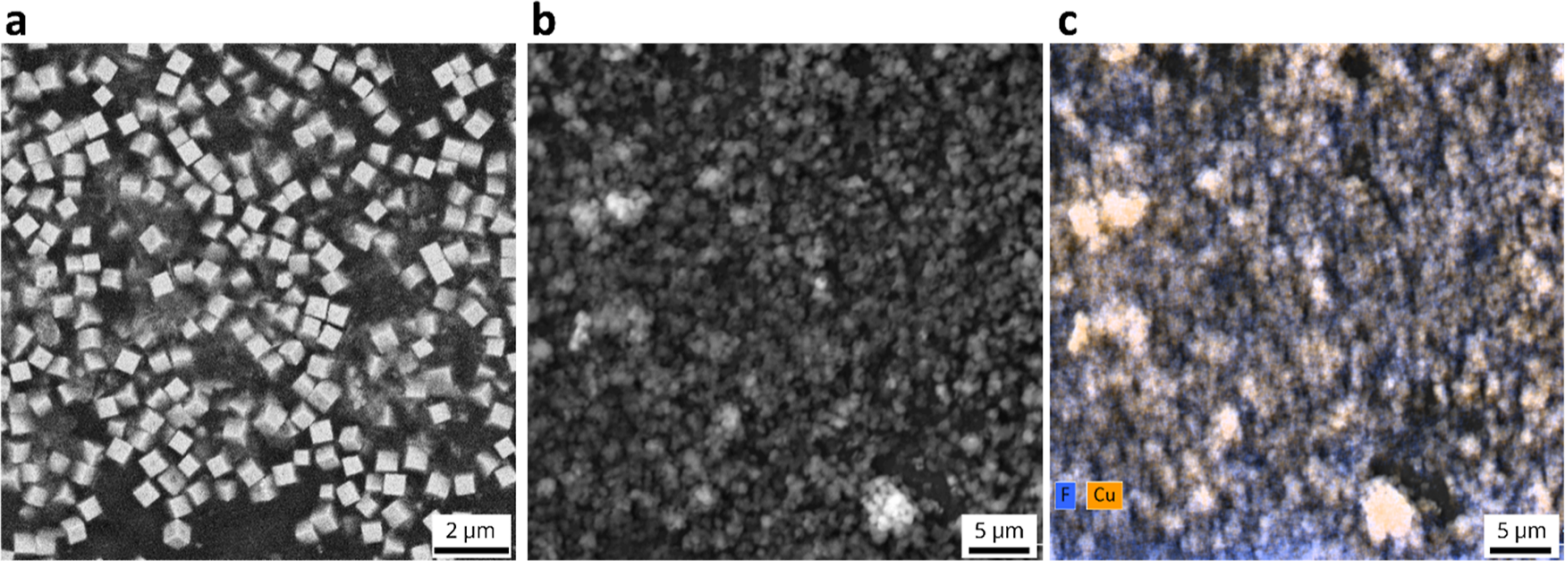
(a). As-prepared Cu_2_O cubes. (b) Cu_2_O cubes and Nafion binder deposited onto a glassy carbon electrode. (c) SEM–EDX map with Cu in orange and F in blue. The Cu_2_O cubes are covered in Nafion binder, limiting the spatial resolution.

Based on these results, we conclude that we have successfully deposited Cu_2_O cubes on a glassy carbon substrate and fabricated electrodes in which the Cu_2_O cubes are fully covered by the Nafion and Sustainion binder. Besides these two electrodes, electrodes without ionomer binder, electrodes with first a Nafion and then a Sustainion layer (Naf-Sus), and electrodes containing first a Sustainion and then a Nafion layer (Sus-Naf) were prepared. These five different electrodes were then used for catalytic performance tests.

### Impact of Ionomers on Catalyst Activity

The effects of the ionomers on the catalytic performance of the Cu_2_O cubes were investigated using five electrodes, containing no ionomer, Nafion, Sustainion, Naf-Sus and Sus-Naf. The measurements were done in triplets (at least three different electrodes were tested for each ionomer) to ensure statistically relevant results. First, the electrodes were reduced to the Cu phase by performing five consecutive CV cycles between +0.3 V and −1.0 V vs RHE at 50 mV/s. The results of the first and fifth cycle are shown in Figure S7a,b, showing Cu_2_O reduction in the first cycle with an onset of −0.1 V vs RHE for all electrodes. The non-Faradaic region at 0.0 V vs RHE, highlighted in gray, is used for subsequent double layer capacitance (DLC) measurements to determine the electrochemically active surface area (ECSA) of the electrodes. It is important to note that the cathodic and anodic scans of the Sus-Naf electrode are not flat and symmetric because the Cl^–^ counterions in the inner Sustainion ionomer layer affect the voltammetric response. This is not the case for the Sustainion-only and Naf-Sus electrodes, as they were ion-exchanged with HCO_3_^–^ before electrolysis to ensure that the catalytic data were not convoluted by ongoing anion exchange as described in previous works.^23,31^

The five electrodes were tested once at four increasingly cathodic potentials from −0.65 V to −1.1 V vs RHE, as shown in Figures S8 and S9. At −0.65 V vs RHE, the total accounted FE is lower than at the other tested potentials. This is possibly because the Cu_2_O was not yet fully reduced. Additionally, the relatively low current densities lead to low product concentrations, which makes accurate product quantification challenging. Although the C_2+_ product selectivity is slightly higher at −1.1 V vs RHE than at −1.0 V vs RHE, the selectivity trends between the electrodes are similar at both potentials. Therefore, −1.0 V vs RHE was chosen for subsequent stability studies on fresh electrodes to better observe the (slower) changes over time and to minimize interference of mass transport limitations in the H-cell.

Subsequently, stability tests were performed at a fixed potential of −1.0 V vs RHE for 20 h. The recorded geometric current densities as a function of electrolysis time are shown in Figure 3a. All electrodes show a decrease in cathodic current density over time, which indicates that they suffer from catalyst deactivation. However, the extent of this deactivation differs per electrode. The largest deactivation is observed for the electrodes with Sustainion and without ionomer, whereas the other three Nafion-containing electrodes have a more stable current density. The periodic dips after 1, 3, and 6 h of electrolysis correspond to measurement interruptions for DLC measurements to quantify the electrochemically active surface area (ECSA) and electrolyte sampling for liquid product quantification using NMR. During these DLC measurements, the applied potential did not exceed +0.2 V vs RHE to prevent bulk copper oxidation, although it cannot be ruled out that some surface oxidation took place in this period. These measurements were carried out nevertheless to obtain more insight into the catalyst stability. Figure 3b shows that all electrodes suffer from a loss of ECSA over time, explaining the catalyst deactivation in Figure 3a. The Sus-Naf electrode shows a much higher ECSA despite similar loadings of Cu_2_O cubes. This is likely because the Cl^–^ counterions in the inner Sustainion layer interfere with the voltammetric response because of anion ad- and desorption,^27,32^ resulting in large error bars as shown in Figure S10 and an overestimation of the Cu ECSA.

**Figure 3:**
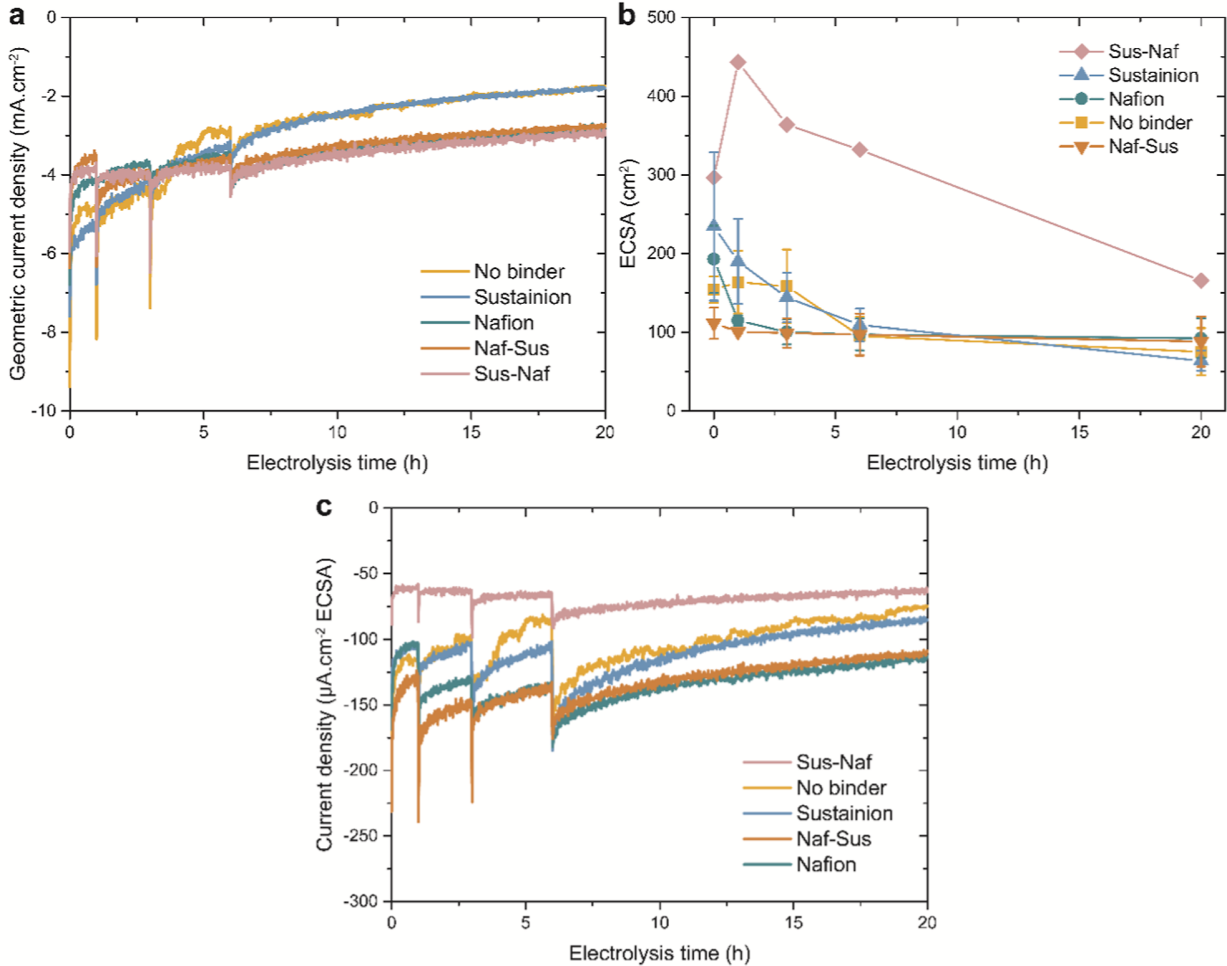
Activity data of 20 h stability tests at −1.0 V vs RHE for the Cu_2_O-based electrodes containing different ionomers. (a) Geometric current density over time. (b) Cu ECSA as determined from double layer capacitance measurements as a function of time. (c) ECSA normalized current density over time, obtained by normalizing for the average ECSA at the start and end of each time interval.

To provide information on the intrinsic activity of the different electrodes, the geometric current densities were normalized by the Cu ECSA as shown in Figure 3c. The Sus-Naf electrode yields the lowest values because of the Cu ECSA overestimation. Interestingly, the electrodes with Sustainion and without ionomer show less cathodic ECSA normalized current densities than the Nafion and Naf-Sus electrodes, with the Nafion electrode showing slightly less cathodic ECSA normalized current densities in the first 3 h of electrolysis before reaching similar values as the Naf-Sus electrode. These results indicate that Sustainion does not affect the intrinsic catalyst activity, whereas Nafion has a promoting effect on the intrinsic activity regardless of the application of a Sustainion overlayer. Possibly, these differences are caused by a difference in product selectivity between the electrodes.

### Impact of Ionomers on Catalyst Selectivity

Besides the catalyst activity, selectivity is an important catalyst performance metric. The Faradaic efficiency (FE) to H_2_, CH_4_, C_2_H_4_ and C_2+_ alcohols of the different electrodes at −1.0 V vs RHE are shown as a function of electrolysis time in Figure 4a–d, respectively. The C_2+_ alcohols include ethanol, n-propanol and allyl alcohol. Their time resolution is less than that of the gas products because of manual electrolyte sampling for liquid product quantification using NMR. Figure S11 shows the CO FE as a function of time, which is similar for all electrodes except for slightly higher values on the Sustainion electrode. Additional catalytic data are provided in Figures S12–S14. Figure S12 shows that the total accounted FE of all electrodes decreases slightly over time. This is likely due to liquid product underestimation caused by evaporation from the catholyte (due to the constant flushing with CO_2_) or by the crossover and oxidation of liquid products at the anode. The latter is evident from liquid products observed with NMR in the anolyte after testing.

**Figure 4:**
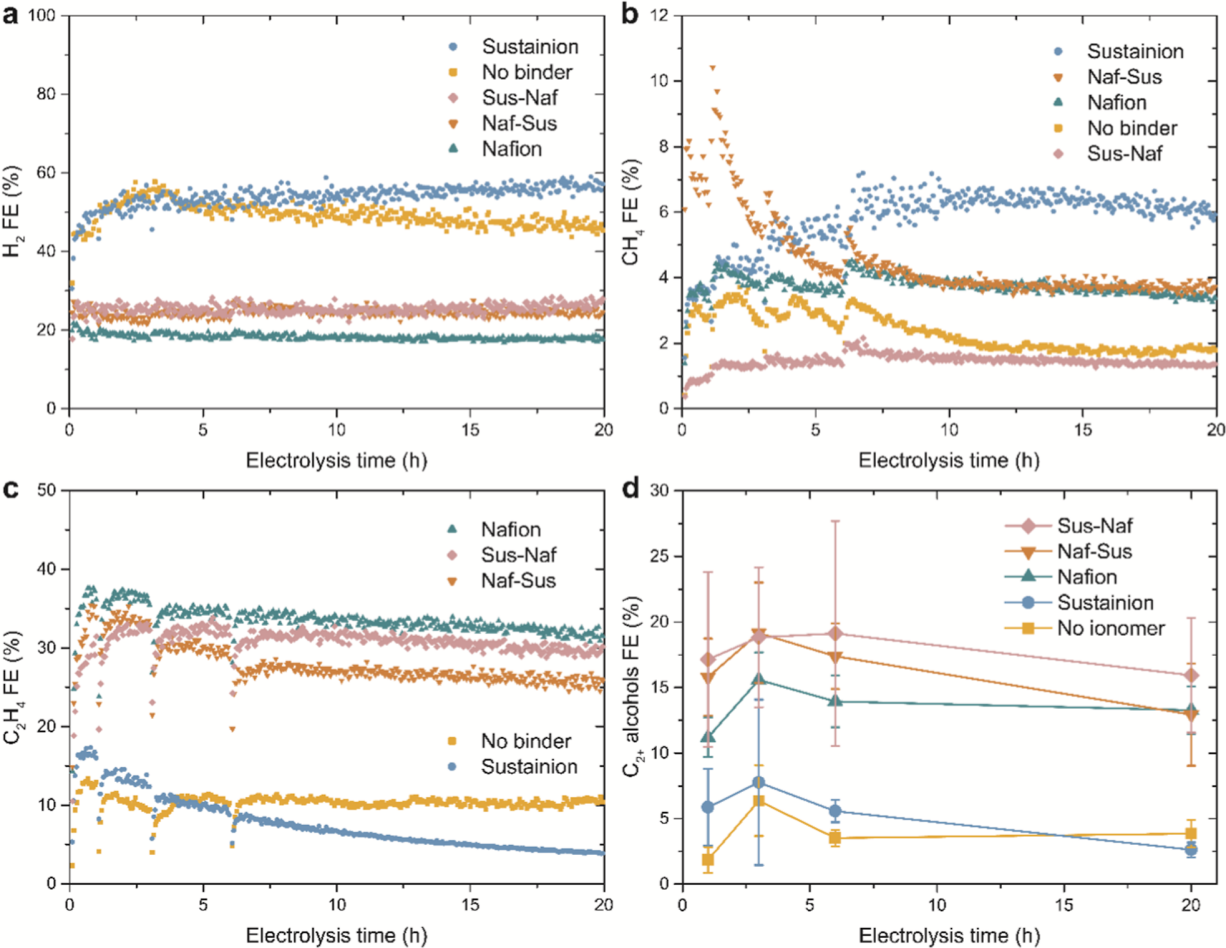
Results of 20 h stability tests at −1.0 V vs RHE for the five Cu_2_O-based electrodes containing different ionomers, showing the FE to (a). H_2_, (b). CH_4_, (c). C_2_H_4_ and (d). C_2+_ alcohols as a function of electrolysis time.

From the results in Figure 4, it becomes clear immediately that the addition of ionomers significantly affects the catalyst selectivity. Especially large differences are observed in the FE to H_2_ as a main byproduct, versus that of the target C_2+_ product molecules like C_2_H_4_, ethanol and n-propanol. On the other hand, the CO and CH_4_ FEs vary less between the different electrodes and do not exceed 10%.

The lowest selectivity, i.e. the highest H_2_ FE and lowest C_2_H_4_ and C_2+_ alcohols FE, is obtained on the electrodes without ionomer coating and with only Sustainion. At first, both of these electrodes have a similar H_2_ FE, but the Sustainion electrode is less stable in terms of selectivity than the electrode without ionomer. In particular, the FEs to C_2_H_4_ and C_2+_ alcohols are at first higher for the Sustainion electrode but decrease more strongly over time. At intermediate electrolysis time, its selectivity shifts to CO and CH_4_, whereas at longer electrolysis time its selectivity is dominated by H_2_. Since the Sustainion electrode also showed the strongest decrease in current density and ECSA in Figure 3, we conclude that it suffers from significant instability issues which have a detrimental effect on both the catalyst activity and selectivity to C_2+_ products over time.

Interestingly, all electrodes containing Nafion show a lower H_2_ FE and a higher C_2+_ product FE than the ones without. This catalytic performance improvement by Nafion can be ascribed to a suppression of the competing HER, as becomes clear upon looking at the H_2_ partial current densities in Figure S13a. Intriguingly, the selectivity improvement is not only due to HER suppression but also due to an improved CO_2_RR activity of the electrodes containing Nafion. In particular, the partial current densities to C_2+_ products are much higher for the electrodes containing Nafion than the ones without, as shown in Figure S13d,e. This suggests that the HER and CO_2_RR compete for the same catalyst active sites and that suppressing one of the reactions promotes the other.

The differences in selectivity are less clear when comparing the Nafion electrode to the electrodes with double ionomer layers. The Sus-Naf and Naf-Sus electrodes have similar H_2_ FE values, whereas those of the Nafion electrode are slightly lower. The Naf-Sus electrode shows a higher CH_4_ FE than the Nafion electrode during the first hours of electrolysis, whereas the Sus-Naf electrode produces the least CH_4_. The Nafion electrode has a slightly higher C_2_H_4_ FE than the Sus-Naf electrode, which is again slightly higher than the Naf-Sus one. Interestingly, the trend in C_2_H_4_ FE is different from that of the C_2+_ alcohols, where the highest FE is observed on the Sus-Naf electrode, followed by the Naf-Sus one. Apparently, the presence of the second ionomer layer affects the ethanol to ethylene ratio.

It is insightful to note that adding Sustainion or Nafion as an overlayer instead of adding it in the catalyst ink does not have much impact on the catalyst selectivity, as shown in Figure S12. This is in contrast to the ionomer layer thickness, which does impact the catalyst selectivity. Figure S13 shows the FE over time of electrodes with half and twice the amount of Nafion. Although adding twice more (2×) Nafion does not affect the catalyst selectivity much, adding half as much (0.5×) Nafion results in a lower C_2+_ product selectivity and higher CH_4_ selectivity after 3 h electrolysis.

These results show that a high degree of copper coverage by Nafion clearly improves the catalyst selectivity to C_2+_ products. Although introducing a second ionomer layer of Sustainion does not improve the overall C_2+_ product selectivity significantly, it does shift the selectivity ratio from ethylene to ethanol. Besides the catalyst activity and selectivity, these results show that also the catalyst stability is affected by the ionomer layers.

### Stability of Cu_2_O-Based Electrodes

To better understand the main causes of the previously observed catalyst deactivation and stability differences for different ionomer layers, the electrodes were characterized using SEM–EDX and XRD after testing. Figure 5 shows SEM–EDX images of the electrodes without ionomer, with Sustainion and with Nafion after catalytic testing for 20 h at high and low magnification. Additional SEM–EDX maps, including those of the Naf-Sus and Sus-Naf electrodes, are provided in Figures S17–S22. The latter two electrodes are not included here because the images are similar to those of the Nafion electrode.

**Figure 5:**
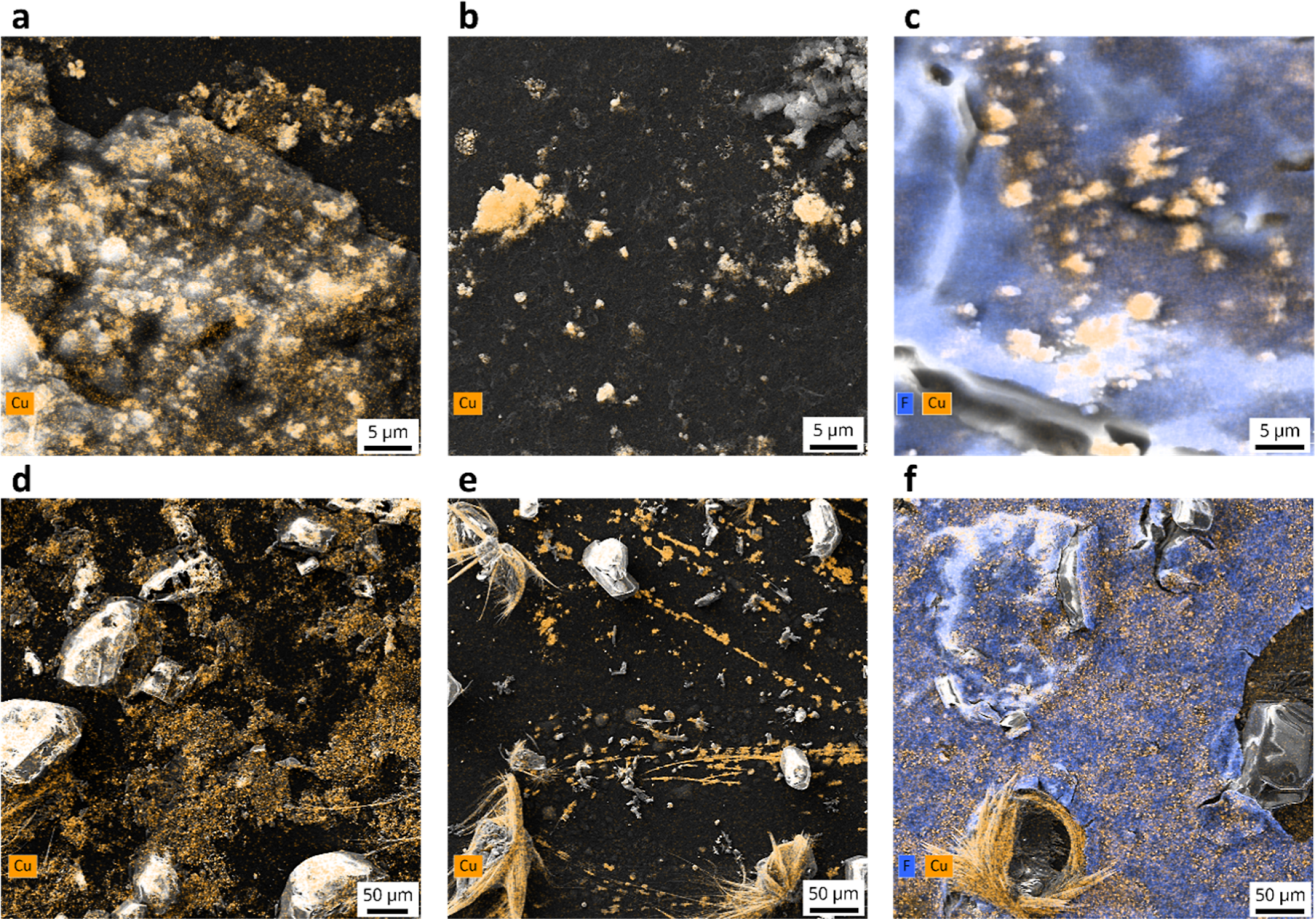
(a). SEM–EDX maps of Cu_2_O cube electrodes after 20 h electrolysis at −1.0 V vs RHE without ionomer (a,d), with Sustainion (b,e) and with Nafion (c,f) at two different magnifications. The EDX maps show Cu in orange and F in blue, with F originating from the PTFE backbone of the Nafion binder.

For all electrodes, pronounced changes are observed in the SEM–EDX images after catalytic testing for 20 h at −1.0 V vs RHE (Figures 5 and S17–S21) compared to the images before testing (Figures 2 and S3–S6) and after only 15 min (Figure S22). The structure of the copper particles, indicated in orange in the EDX maps, clearly deviates from the original cubic shape in all cases. This loss of cubic shape is fast and takes place almost fully in the first 15 min of electrolysis. Clear differences are observed between the electrodes with different ionomer layers. Without ionomer coating, dendritic structures larger than the 581 nm of the original cubes have formed after 20 h electrolysis. This indicates that nanoclustering and agglomeration of Cu occurred during the catalysis, as commonly observed for Cu-based electrodes.^5^ Nevertheless, smaller Cu structures are also observed and the Cu is still relatively well dispersed on the electrode. In contrast, for the Sustainion electrode, copper dendrites have already been formed within 15 min of electrolysis. After 20 h at −1.0 V vs RHE, the copper dendrites have grown significantly, resulting in large parts of the electrode surface being devoid of copper. This is despite Sustainion ionomer still being present, visible as a region of intermediate contrast prone to charging in Figure S19b. This suggests that Sustainion increases the copper mobility during CO_2_RR, resulting in the formation of large dendritic agglomerates.

For the Nafion electrode, roughened spherical particles similar in size to the original cubes are detected even after 20 h electrolysis. However, larger agglomerates composed of several such particles, as well as large copper dendrites are also observed. Nafion has remained attached to most of the electrode surface, as evidenced by the presence of F originating from the PTFE backbone in blue. Interestingly, large copper dendrites (see Figure 5f) have formed at the same place where Nafion has detached. All in all, copper restructuring is evident, but especially upon comparison to the other two electrodes, we find that Nafion seems to stabilize the copper structure by mitigating agglomeration. This stabilizing effect is also observed for the Naf-Sus and Sus-Naf electrodes in Figures S20 and S21, as evidenced by the presence of individual particles similar in size to the original Cu_2_O cubes.

Besides the copper structure, Figure 5 shows regions of high contrast which suffered from charging during SEM imaging. The EDX maps in Figures S17–S21 indicate that these regions correlate well to the presence of K (and O, not shown). Likely, these are (bi)­carbonate salt precipitates, which form due to a combination of CO_2_ and a high local pH during CO_2_RR. Indeed, XRD measurements in Figure S23 indicate that potassium bicarbonate is present on all electrodes after catalytic testing, despite thorough rinsing with Milli-Q water before the XRD measurements. The deposition of potassium bicarbonate provides another explanation for the loss of total current density and ECSA in Figure 3 by blocking parts of the electrode surface.

These stability results help to explain the observed activity and selectivity trends over time. The electrode with Sustainion starts with a higher C_2+_ product selectivity than the electrode without ionomer, indicating that at first the Sustainion ionomer slightly improves the catalyst selectivity. However, a significant decay in activity and selectivity is observed. This is related to severe copper restructuring into large dendritic agglomerates in combination with bicarbonate precipitation. On the other hand, Nafion shows a smaller decay in activity over time. This can be explained by a stabilizing effect of Nafion on the copper structure, leading to reduced agglomeration and possibly mitigating bicarbonate precipitation via electrochemical exclusion of bicarbonate anions. The slight decrease in catalyst activity over time can be explained by Nafion detachment resulting in the formation of large copper dendrites protruding from the ionomer coating. The relatively stable selectivity of the Nafion-containing electrodes in Figure 5 contrasts with the fact that the copper particles change from a cubic to a roughened spherical shape. However, Figure S21 shows that this reshaping takes place already within the first 15 min of electrolysis, hence it does not impact the stability trends in a time frame of 20 h of electrolysis. Thus, these results reveal that the structural/morphological changes in Cu_2_O electrodes during CO_2_RR have a minimal impact on the catalyst’s selectivity over 20 h, especially when coated with a suitable ionomer.

### Effect of First Ionomer Layer on the Catalyst Microenvironment: Nafion vs Sustainion vs No Ionomer

To better understand the trends in catalytic performance for different ionomer coatings, it is important to discuss how the ion transport within these layers is regulated. Figure 6a depicts the ion transport in the absence of an ionomer coating during the CO_2_RR. For clarity, the flow of CO_2_ toward and the flow of products away from the electrode are omitted. During electrolysis, protons are consumed to form H_2_O and/or H_2_O is consumed to form OH^–^. With bicarbonate ions present at the interface, these ions act as proton donors and buffer the local pH by reacting with OH^–^ to form CO_3_^2–^ ions. It should be noted that for the used concentration of 0.1 M KHCO_3_, likely a combination of water-mediated and bicarbonate-mediated reduction takes place.^16^ In addition, K^+^ ions are present at the electrochemical interface to ensure electroneutrality, as well as to stabilize negatively charged reaction intermediates, thus facilitating the water-mediated HER and CO_2_RR.^15^

**Figure 6:**
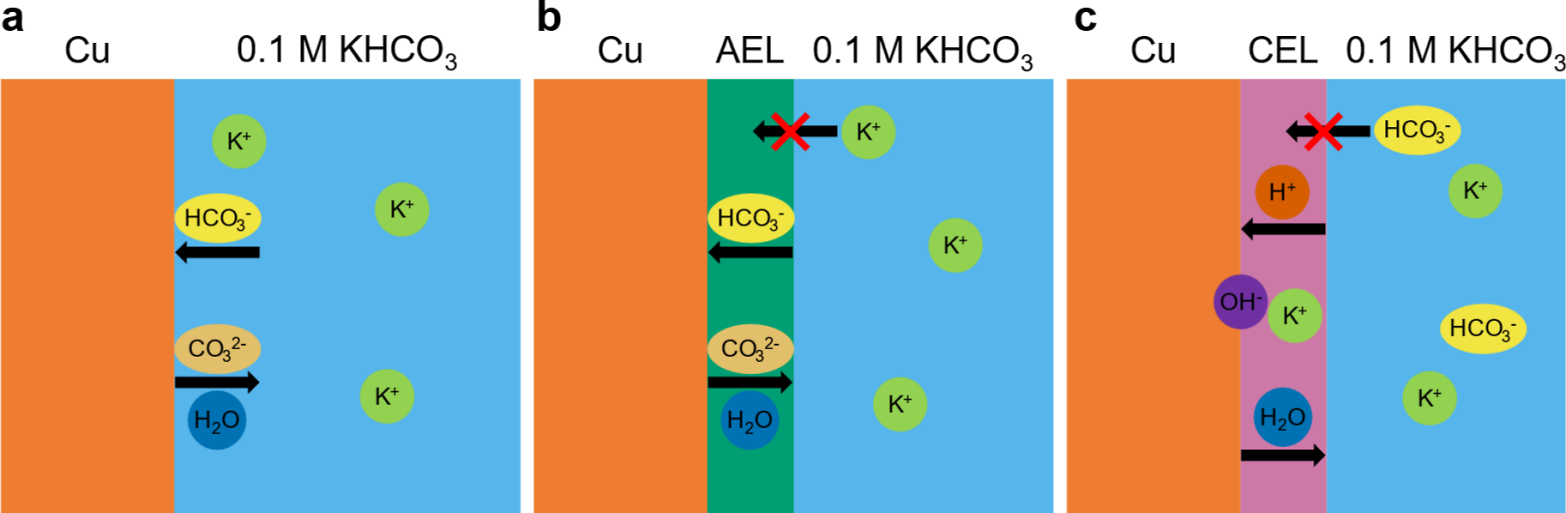
Schematics showing the flow of ions during CO_2_ electrolysis in the presence of (a). No ionomer coating, (b). With an AEL of Sustainion and (c). With a CEL of Nafion. For clarity, the flow of CO_2_ toward and the flow of CO_2_RR products away from the electrodes are omitted as they are the same for all electrodes.

This situation changes upon the addition of an anion-exchange layer (AEL) of Sustainion. First, it is good to note that we do not expect the flow of CO_2_ to be impeded by the ionomers layers of either Sustainion or Nafion, given that the solubility of CO_2_ is reported to be higher in bulk Sustainion membranes and similar for bulk Nafion membranes with respect to aqueous electrolytes.^23^ The positively charged imidazolium functional groups of Sustainion will repel positively charged K^+^ ions, whereas there is an enrichment of (bi)­carbonate anions because of Donnan exclusion effects.^19,24^ Given that the catalyst activity and selectivity of the Sustainion electrode are similar to that of the electrode without ionomer coating at the start of electrolysis, we do not expect these concentration differences to be sufficiently large to significantly affect the catalyst performance. This makes sense considering that the flow of (bi)­carbonate anions is not impeded by the presence of a Sustainion layer. Rather, the major difference between the electrodes with and without Sustainion is more deactivation of the Sustainion electrode during electrolysis, as clear from a greater loss of ECSA, current density and C_2+_ product selectivity. A possible explanation is more (bi)­carbonate precipitation in the Sustainion layer, because the (bi)­carbonate to water ratio is higher in this layer than in the bulk electrolyte.

The situation is again different when a cation exchange layer (CEL) of Nafion is present. The negatively charged sulfonate functional groups repel (bi)­carbonate anions, and increase the abundance of K^+^ ions. Possibly, the enrichment of K^+^ ions plays a role in the higher ECSA normalized current densities (Figure 3c) because of stabilization of negatively charged reaction intermediates, although it is also possible that the higher catalyst activity can be explained solely by the higher selectivity to many-electron C_2+_ products. The suppressed HER is likely due to exclusion of proton-donating bicarbonate ions, with water acting as the proton donor instead. Given that Nafion lowers the local water concentration with respect to the aqueous electrolyte,^33^ water-mediated HER will be suppressed by the Nafion layer as well. Water acting as the proton donor results in the formation of OH^–^ ions near the copper surface during electrolysis. Because the CEL prevents OH^–^ and HCO_3_^–^ transport, the bicarbonate ions can only buffer the local pH via proton donation through the CEL. This leads to the accumulation of OH^–^ ions near the copper surface, resulting in an increased local pH. This also contributes to the HER suppression and promotion of CO_2_RR selectivity by Nafion (Figure 4).

### Effect of an Extra Ionomer Layer: Naf-Sus and Sus-Naf

Looking at the electrodes containing two stacked ionomer layers, the Naf-Sus electrode performs similar to the Nafion one. This is expected given that the inner ionomer layer is the same, and hence no major differences are expected for the ion concentrations and pH in the catalyst microenvironment as shown in Figure 7a. The slightly higher H_2_ FE on the Naf-Sus than the Nafion electrode could be due to a slightly more acidic microenvironment in the Naf-Sus case. During electrolysis in the Nafion-only electrode, protons are reduced at the cathode surface and replenished by a combination of H^+^ and K^+^ ions from the bulk electrolyte. However, the extra AEL of the Naf-Sus electrode prevents K^+^ ions from reaching the CEL. This results in relatively more H^+^ counterions and a slightly lower local pH which in turn lowers the catalyst selectivity.

**Figure 7:**
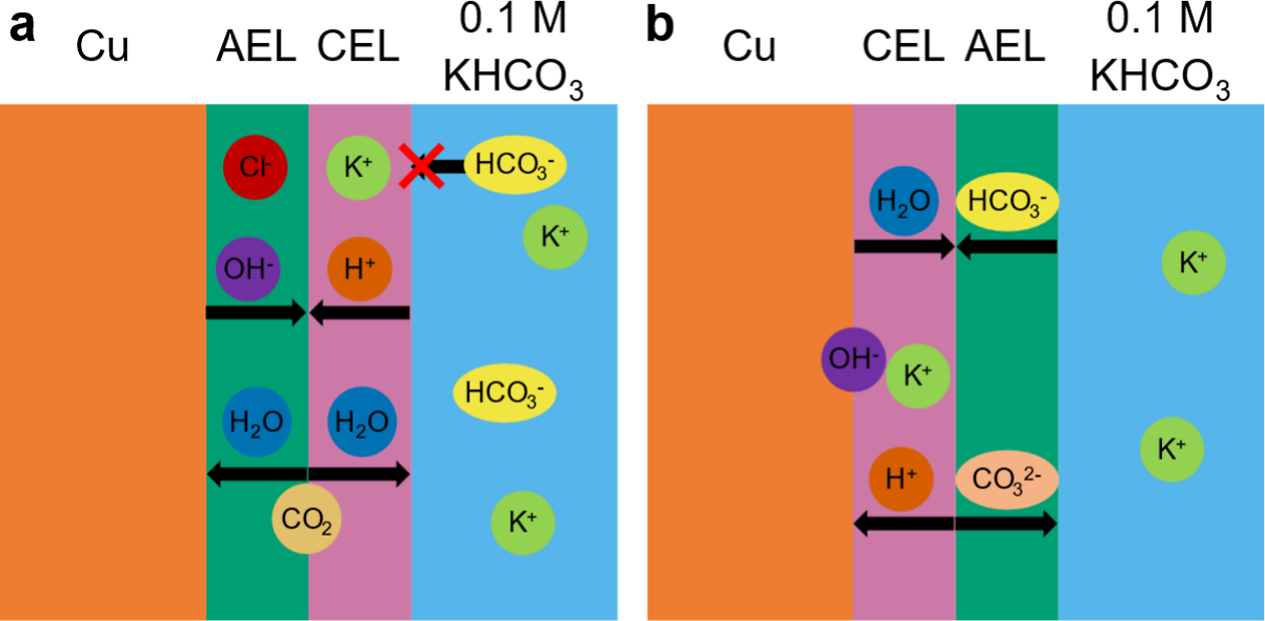
Schematics showing the flow of ions in the presence of (a). Naf-Sus (CEL-AEL) and (b). Sus-Naf (AEL-CEL) ionomer coatings.

Another intriguing difference is the higher ethanol to ethylene ratio of the Naf-Sus electrode than the Nafion one. Surprisingly, the Sus-Naf electrode performs similar in selectivity to the Nafion and Naf-Sus ones, and also shows a higher ethanol to ethylene ratio than the Nafion electrode. These results indicate that the inner ionomer layer does not govern the catalyst selectivity exclusively, but that also the outer ionomer layer plays an important role. The ethanol to ethylene ratio trend suggests that the bilayer electrodes feature a higher *CO coverage on Cu,^34^ higher local pH^35^ or different (asymmetric) surface structures^7,12,36^ than for Nafion only. However, identifying the main cause of this selectivity shift requires more detailed in situ and/or mechanistic investigations. Given that this effect is observed for both double ionomer layers, possibly the increased ionomer layer thickness or higher residence time of relevant reaction intermediates might play a role,^36^ although this effect is not observed when adding twice as much Nafion (Figure S16).

### Effect of the Counteranion in the Inner Sustainion Layer of Sus-Naf

It is important to note that the Sus-Naf electrode is not ion-exchanged, and therefore contains Cl^–^ instead of HCO_3_^–^ counterions in the AEL. The ion exchange was not performed since the counterions are expected to remain within the AEL during electrolysis, as the CEL prevents their exchange with the bulk electrolyte as shown in Figure 7b. This is in contrast to the Sustainion-only and Naf-Sus electrodes, in which the Sustainion layer is in direct contact to the bulk 0.1 M KHCO_3_ electrolyte.

To examine the effect of the counterion in the Sustainion layer, additional Sus-Naf electrodes were prepared in which the inner Sustainion layer was ion-exchanged with KHCO_3_ or KOH before application of the Nafion overlayer. The catalytic performance data are included in Figures S24–S27. Although the experiments were conducted only once, it is clear that the KHCO_3_ and KOH exchanged Sus-Naf electrodes exhibit a higher H_2_ and lower C_2_H_4_ FE over time than the Sus-Naf electrode with Cl^–^ counterions. Additionally, their total current density is more unstable and similar to the Sustainion electrode. These results indicate that the counteranions in the inner Sustainion layer are trapped by the Nafion overlayer and that the Nafion overlayer also prevents anion exchange with the bulk 0.1 M KHCO_3_ electrolyte.

Given that the Nafion overlayer allows the counteranions in the Sustainion layer to remain during electrolysis, we have obtained a set of electrodes that allow us to examine anion effects on the catalytic performance of Cu. Similar to the Nafion-only electrode, a low bicarbonate concentration near the copper surface suppresses the competing HER, which is beneficial for the catalyst activity and selectivity to C_2+_ products. In addition, the exclusion of bicarbonate ions near the copper surface mitigates the formation of (bi)­carbonate deposits, limiting catalyst deactivation. These results clearly show that ionomer layers have a strong influence on the microenvironment and consequently the catalytic performance of Cu via regulation of the selective transport of ions.

## Conclusions

In this work, we have shown that ionomer layers significantly affect the performance of copper-based electrodes in CO_2_ electroreduction (CO_2_RR) by regulating the selective transport of ions. When a cation-exchange layer (Nafion) is used, regardless of the presence of an additional inner or outer anion-exchange layer (Sustainion), the competing hydrogen evolution reaction is suppressed and the partial current density and Faradaic efficiency to value-added C_2+_ products are increased. This is due to exclusion of bicarbonate ions from the catalyst microenvironment by Nafion, since bicarbonate ions lower the CO_2_RR selectivity by acting as proton donors and buffering the local pH. Interestingly, introducing an additional inner or outer Sustainion layer caused a selectivity shift from ethylene to ethanol. Sustainion-only electrodes showed a high deactivation rate because of Cu restructuring into large dendrites and precipitation of potassium bicarbonate on the electrode. Although deactivation is also observed for Nafion-containing electrodes, individual Cu particles were still present after 20 h of electrolysis. This reveals the ability of Nafion to limit Cu mobility and restructuring, thereby leading to a more stable catalytic activity. Thus, our work demonstrates that applying (multiple) ionomer layers is an effective means of regulating the transport of ions between the ionomer layers and the bulk electrolyte. The insights obtained underline the importance of achieving control over the catalyst microenvironment for developing electrodes with high CO_2_ reduction selectivity and stability.

## Supplementary Material



## Notes

Electrochemical cell schematic, detailed NMR quantification, additional SEM–EDX images and electrocatalytic data (PDF)

## Abbreviations

AEL: anion-exchange layer
CEL: cation-exchange layer
DMSO: dimethyl sulfoxide
DLC: double layer capacitance
CO_2_RR: CO_2_ reduction reaction
ECSA: electrochemical surface area
EDX: energy-dispersive X-ray spectroscopy
FE: Faradaic efficiency
GC: gas chromatograph
GCE: glassy carbon electrode
HER: hydrogen evolution reaction
NMR: nuclear magnetic resonance
RHE: reversible hydrogen electrode
SEM: scanning electron microscope
XRD: X-ray diffraction.
