# Supplementary material for: Ionomer-Modulated Electrochemical Interface Leading to Improved Selectivity and Stability of Cu2O‑Derived Catalysts for CO2 Electroreduction
Source: ACS Catal. 2025 May 23;15(11):9695–705. doi: 10.1021/acscatal.5c01614 (PMC12150392; doi:10.1021/acscatal.5c01614)
Supplement: Supplementary file 1 [file cs5c01614_si_001.pdf]

Supporting Information

Ionomer-modulated electrochemical interface leading to improved selectivity and stability of Cu_2O-derived catalysts for CO_2 electroreduction

Matt L.J. Peerlings[1], Maaike E.T. Vink-van Ittersum[1], Jan Willem de Rijk[1], Petra E. de Jongh[1], Peter Ngene[1]*

1. Materials Chemistry and Catalysis, Debye Institute for Nanomaterials Science, Utrecht University, 3584 CG Utrecht, The Netherlands

* Email: P.Ngene@uu.nl

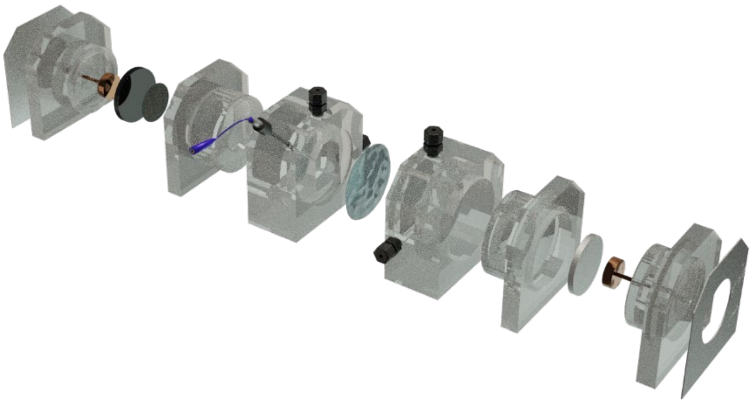

Figure S1. Custom-made H-type electrochemical cell used for catalytic tests. From left to right: glassy carbon electrode current collector containing the Cu_2O catalyst, cathode compartment with Ag/AgCl reference electrode, Fumasep FAA-3-PK-130 anion exchange membrane, anode compartment, and commercial IrO_2-based counter electrode pressed against a glassy carbon electrode current collector.

Product quantification using NMR

Products remaining in the liquid phase were analyzed using a 400 MHz VNMRS-400 Varian NMR. Samples tubes were filled with a mixture of 500 µL electrolyte and 100 µL internal standard solution containing 10 mM DMSO and 50 mM phenol in D_2O. This resulted in a concentration of 1.67 mM DMSO and 8.33 mM phenol in each tube. By comparing the product and internal standard signals A_x, the product concentrations inside the NMR tubes $C_{x,\ in\ NMR\ tube}$ were obtained. For formate quantification, the phenol peak corresponding to 2 hydrogen atoms was used as internal standard:

$$C_{HCOOH,in\ NMR\ tube}\ (mM) = 8.33 * \frac{A_{HCOOH}}{1} * \frac{2}{A_{phenol}}$$

For the other products, the peak areas A_x corresponding to 3 hydrogen atoms and the DMSO internal standard (6 hydrogen atoms) were used for quantification:

$$C_{x,in\ NMR\ tube}\ (mM) = 1.67 * \frac{A_x}{3} * \frac{6}{A_{DMSO}}$$

These values were used to calculate the moles of product n_x in each NMR tube:

$$n_{x,in\ NMR\ tube}\ (mol) = C_{x,in\ NMR\ tube} * 600 * 10^{-9}$$

The total amount of product formed was calculated by accounting for the electrolyte volumes inside the NMR tube (0.5 mL) and inside the electrochemical cell ($V_{catholyte}$, 17 mL):

$$n_{x,total} = n_{x,in\ NMR\ tube} * \frac{V_{catholyte}}{0.5}$$

Multiple electrolyte samples of 1 mL were taken during each catalytic test and replaced with fresh 0.1 mM $KHCO_3$. This was accounted for:

$$n_{x,corrected} = n_x - n_{x,previous\ sample} * \frac{16}{17}$$

With n_x and $n_{x,\ previous\ sample}$ the molar amounts of product calculated based on the NMR peak areas of the current and previous electrolyte sample, respectively. The corrected amount of product $n_{x,\ corrected}$ was subsequently used to calculate the Faradaic Efficiency of each product *x* (FE_x) according to the formula:

$$FE_x\ (\%) = \frac{n_{x,corrected} * n_e * \mathrm{F}}{i_{tot} * t}$$

With n_e the number of electrons transferred for each product, *F* the Faraday constant (96 485 C mol^{-1}), i_{tot} the average current in *A* and *t* the electrolysis time in *s*.

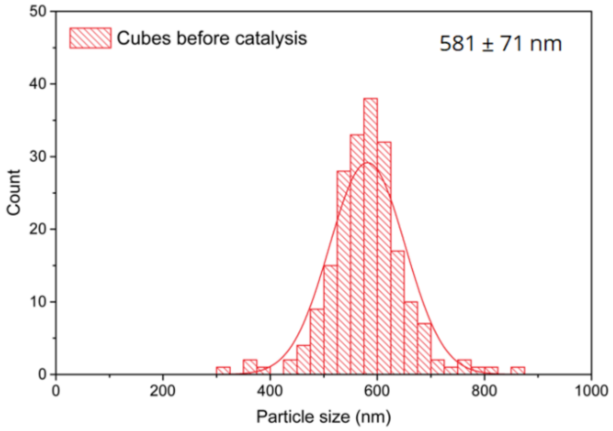

Figure S2. Histogram showing the particle size distribution of the Cu_2O cubes prepared via a colloidal synthesis method. The given sizes correspond to the edge length of individual cubes, being 581 nm on average with a standard deviation of 71 nm.

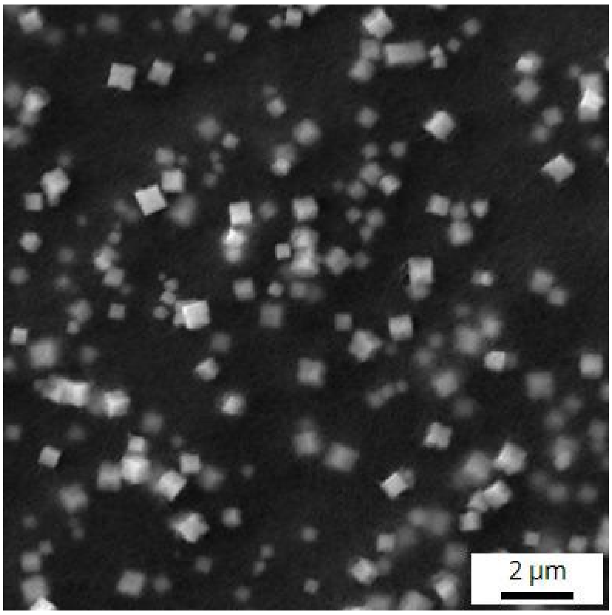

Figure S3. a. Cu_2O cubes without binder deposited onto glassy carbon electrode.

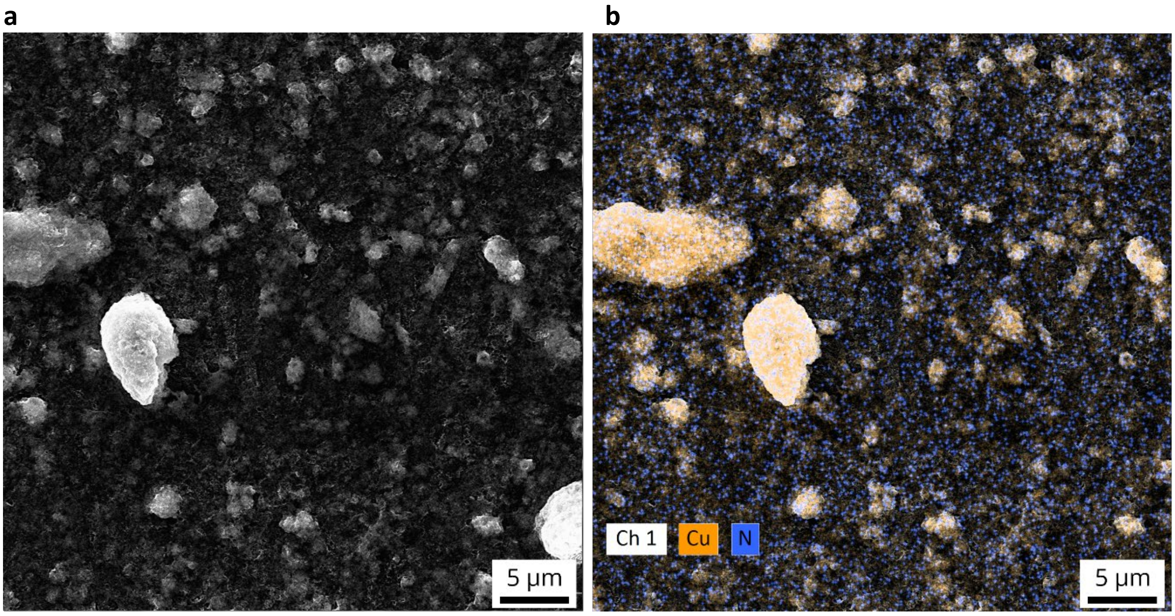

Figure S4. a. Cu_2O cubes and Sustainion binder deposited onto glassy carbon electrode. **b.** SEM-EDX map with Cu in orange and N in blue. The weak N signal originates from the two nitrogen atoms of the imidazolium functional group. The Cu_2O cubes are covered in Sustainion binder, limiting the spatial resolution.

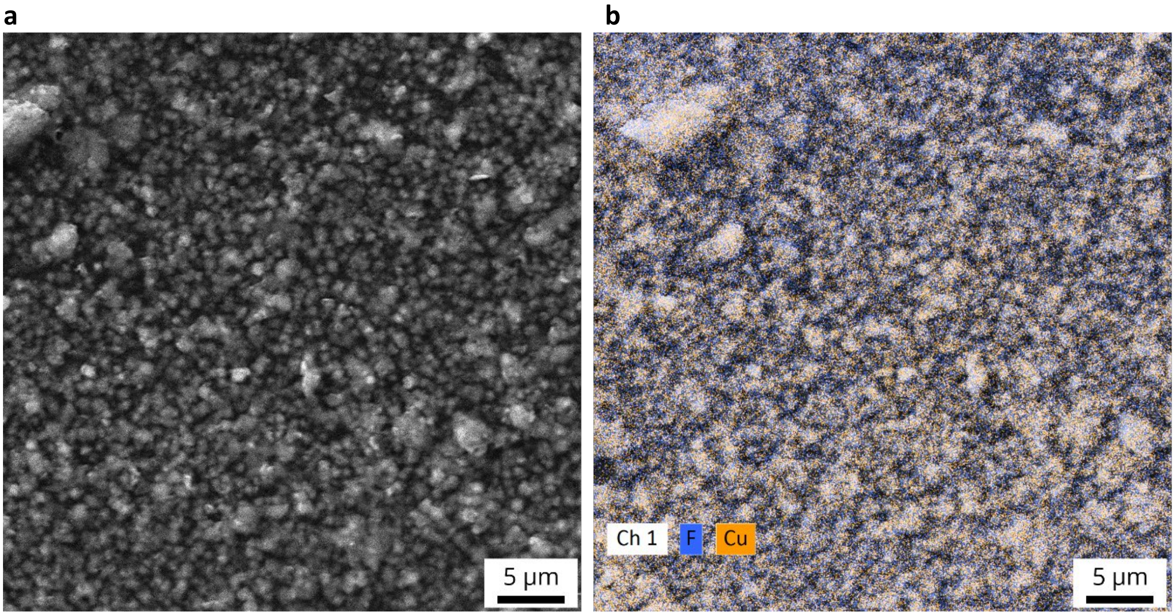

Figure S5. a. SEM-image of Cu_2O-Naf-Sus electrode. **b.** SEM-EDX map with Cu in orange and F in blue.

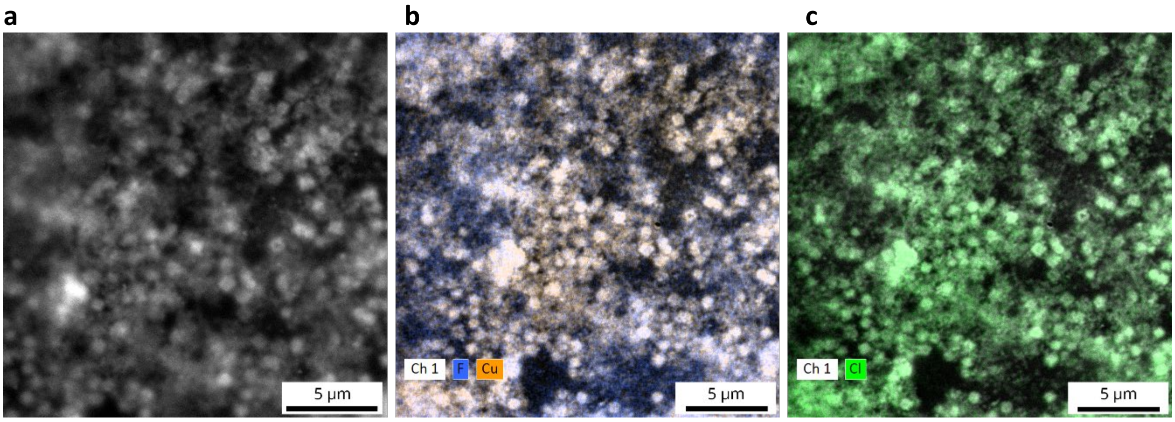

Figure S6. a. SEM-image of Cu_2O-Sus-Naf electrode. **b.** SEM-EDX map with Cu in orange and F in blue. **c.** SEM-EDX map of Cl in green, showing the presence of Cl^- counterions in the inner Sustainion layer.

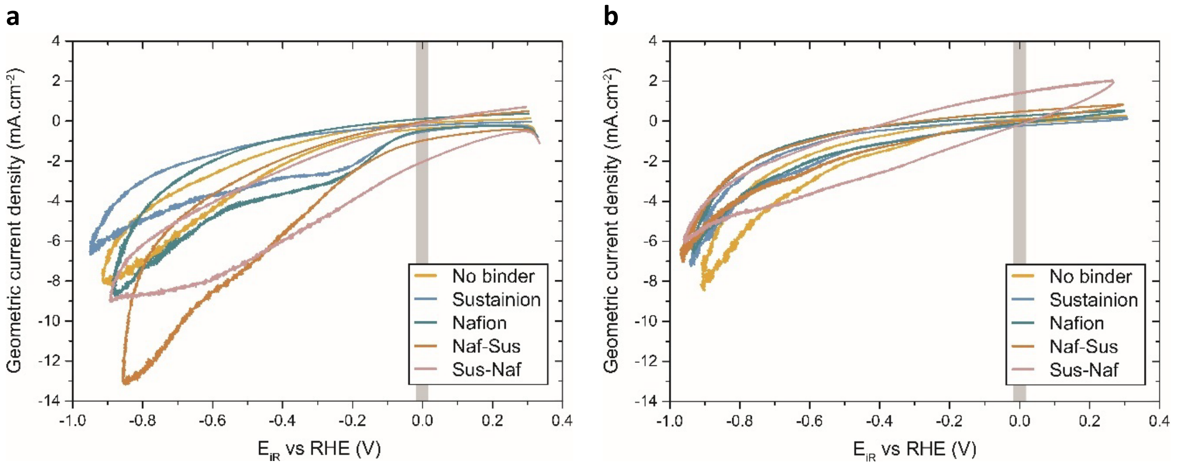

Figure S7. a. First and **b.** fifth cyclic voltammetry cycles of the Cu_2O electrodes with different ionomer layers from +0.3 V to -1.0 V vs RHE at 50 mV/s. A sharp decrease in cathodic current is visible upon cycling, indicating cuprous oxide reduction. The current width at 0 V vs RHE, highlighted in gray, is used for double layer capacitance measurements to determine the electrochemical surface area.

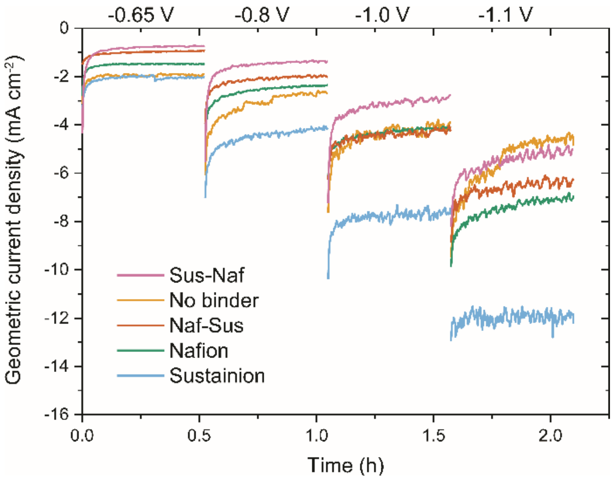

Figure S8. Geometric current density as function of electrolysis time for the electrodes tested at four increasingly cathodic potentials from -0.65 V to -1.1 V vs RHE for half an hour each.

Faradaic Efficiency at -0.65 V vs RHE

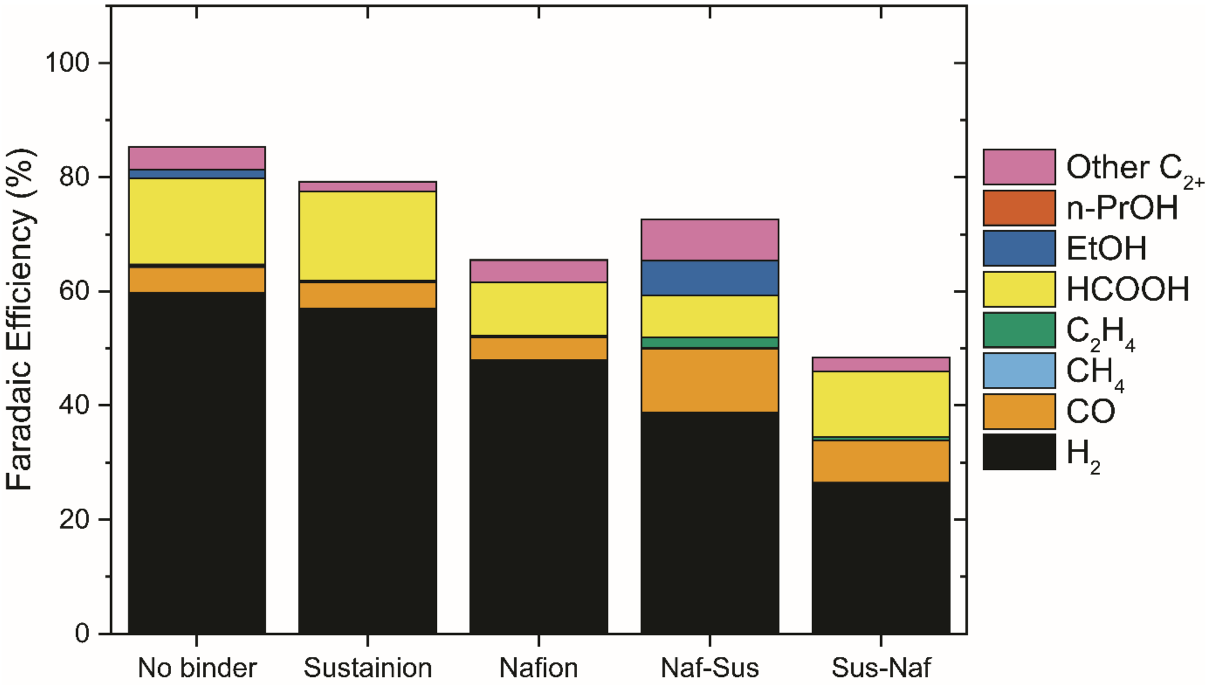

Figure S9a. Faradaic Efficiency of all accounted products at -0.65 V vs RHE for the Cu_2O electrodes with different ionomer layer(s). Other C_{2+} products include ethane, acetate, ethylene glycol, acetone, ethanal and allyl alcohol.

Faradaic Efficiency at -0.8 V vs RHE

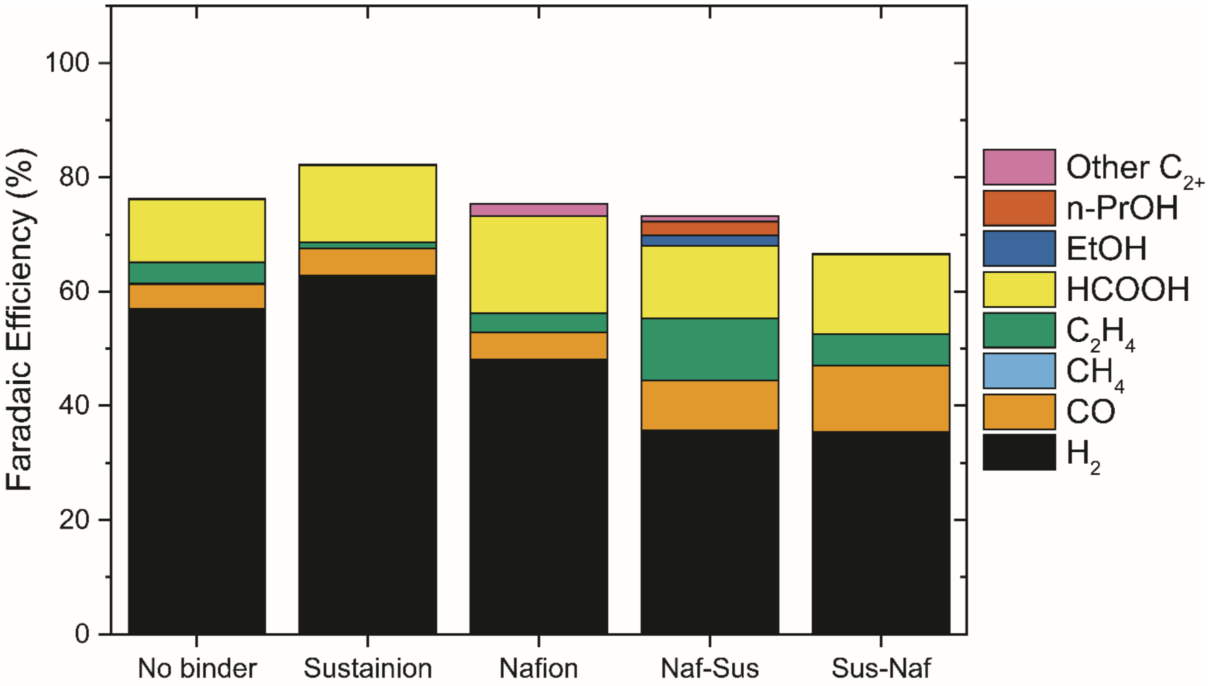

Figure S9b. Faradaic Efficiency of all accounted products at -0.8 V vs RHE for the Cu_2O electrodes with different ionomer layer(s). Other C_{2+} products include ethane, acetate, ethylene glycol, acetone, ethanal and allyl alcohol.

Faradaic Efficiency at -1.0 V vs RHE

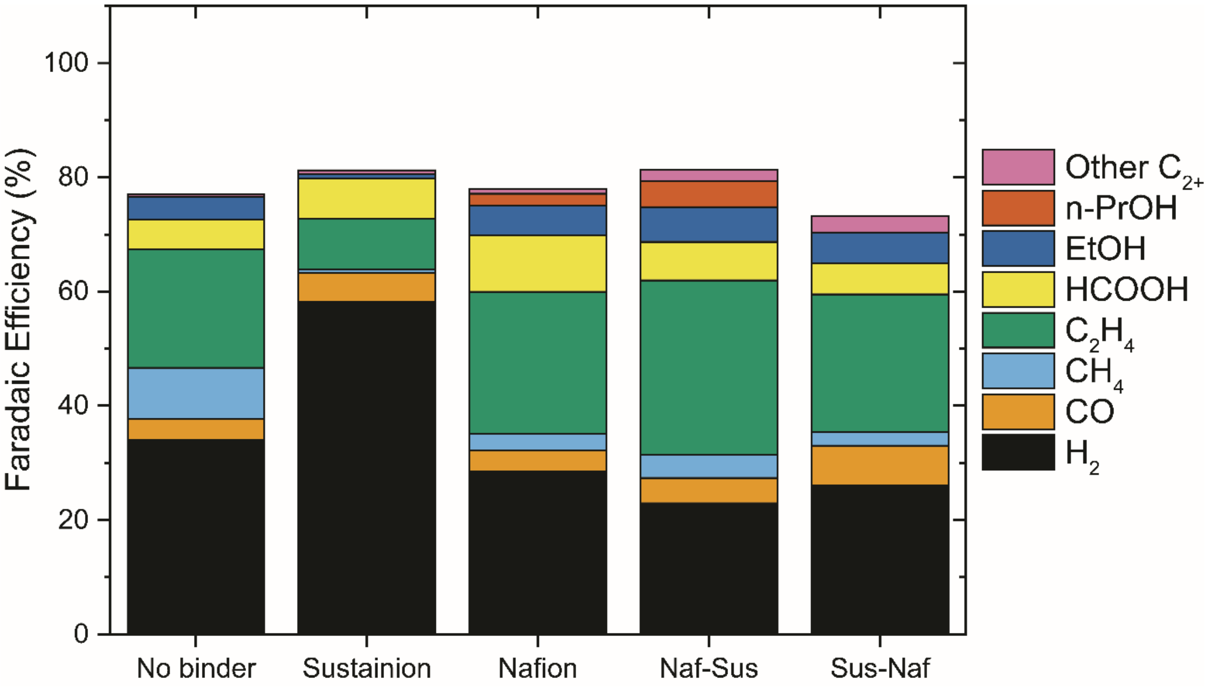

Figure S9c. Faradaic Efficiency of all accounted products at -1.0 V vs RHE for the Cu_2O electrodes with different ionomer layer(s). Other C_{2+} products include ethane, acetate, ethylene glycol, acetone, ethanal and allyl alcohol.

Faradaic Efficiency at -1.1 V vs RHE

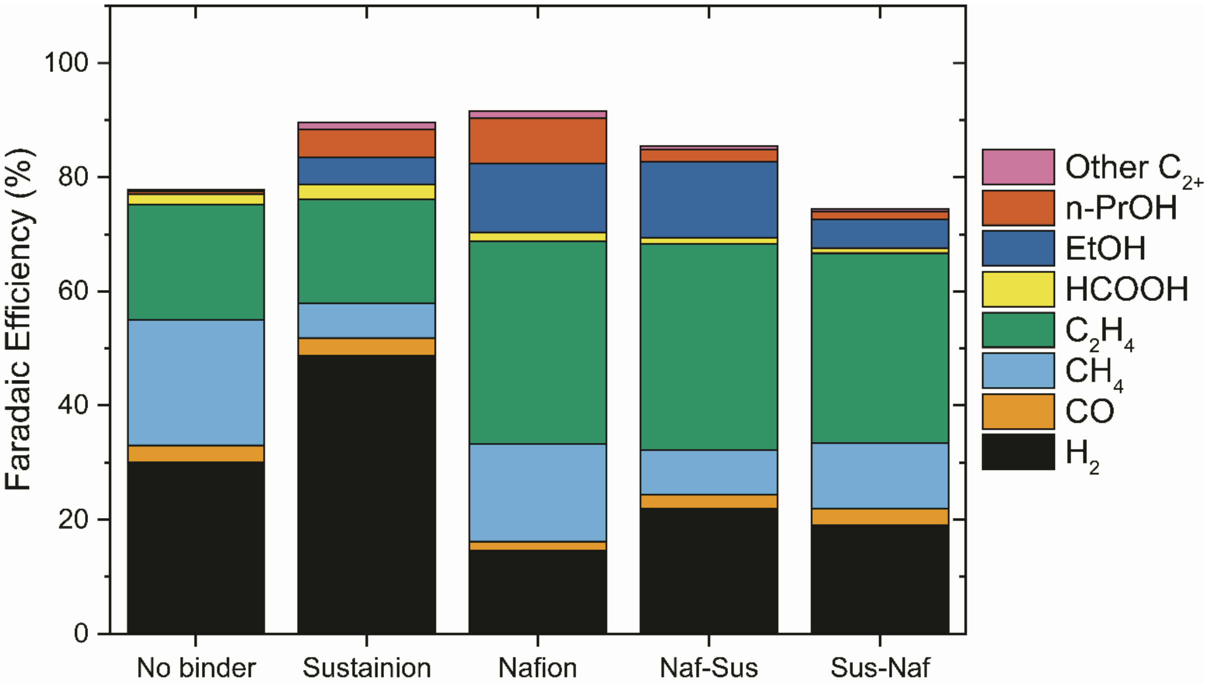

Figure S9d. Faradaic Efficiency of all accounted products at -1.1 V vs RHE for the Cu_2O electrodes with different ionomer layer(s). Other C_{2+} products include ethane, acetate, ethylene glycol, acetone, ethanal and allyl alcohol.

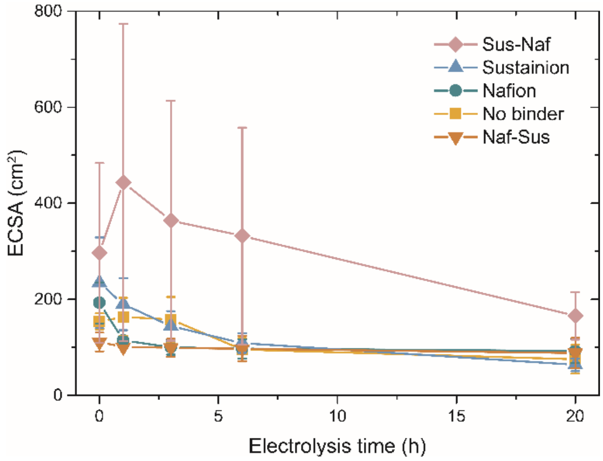

Figure S10. Cu ECSA as determined from double layer capacitance measurements as a function of time, showing large error bars for the Sus-Naf electrode due to Cl^- ions preventing an accurate determination.

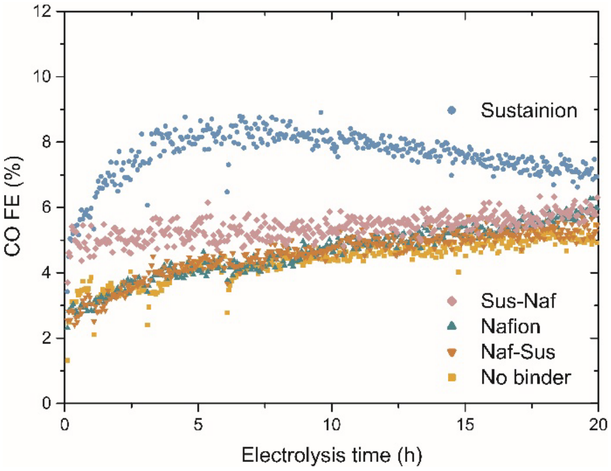

Figure S11. CO FE as function of time for the different electrodes.

Cu_2O no binder

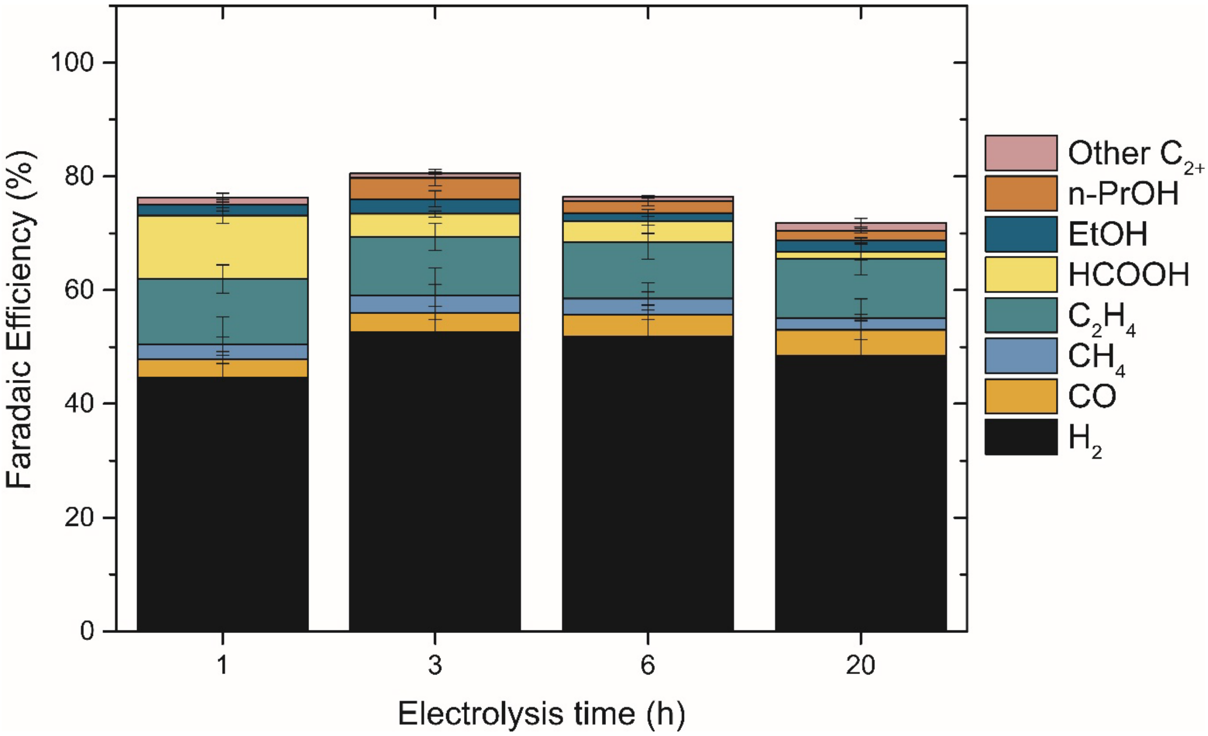

Figure S12a. Faradaic Efficiency of all products as function of electrolysis time for the Cu_2O electrode without ionomers. Other C_{2+} products include ethane, acetate, ethylene glycol, acetone, ethanal and allyl alcohol.

Cu_2O with Sustainion

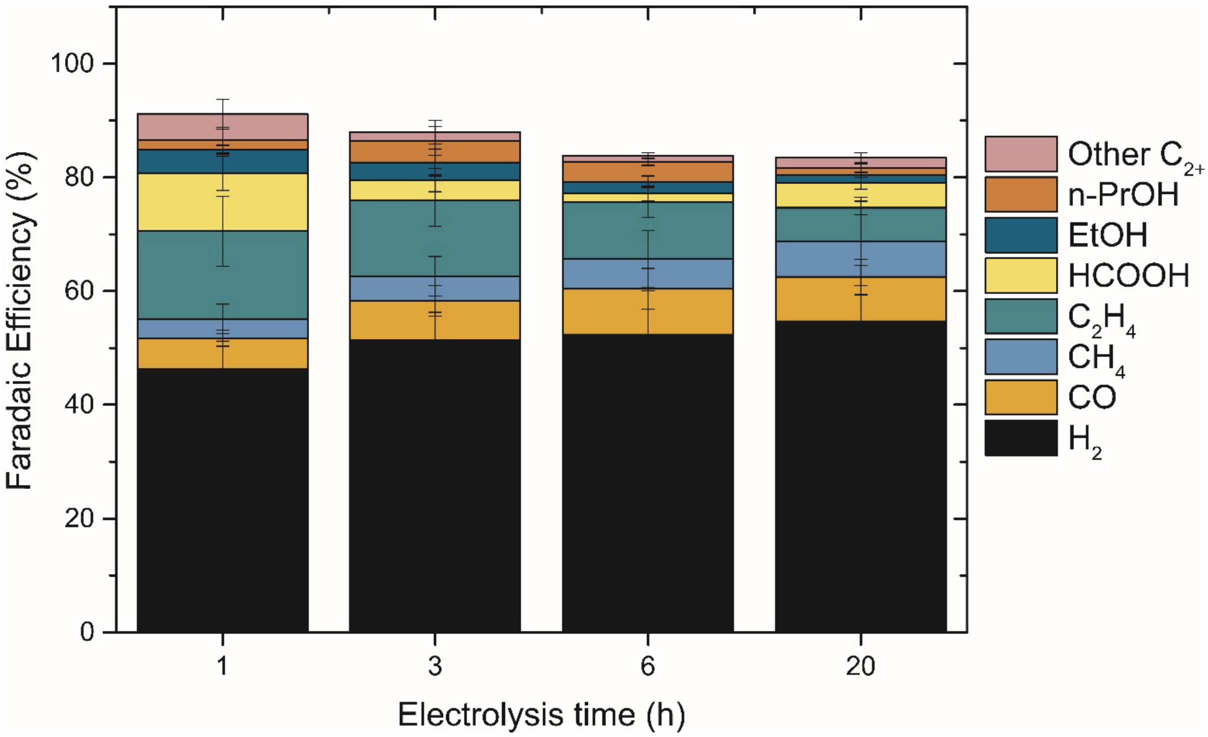

Figure S12b. Faradaic Efficiency of all products as function of electrolysis time for the Cu_2O electrode with Sustainion ionomer. Other C_{2+} products include ethane, acetate, ethylene glycol, acetone, ethanal and allyl alcohol.

Cu_2O with Nafion

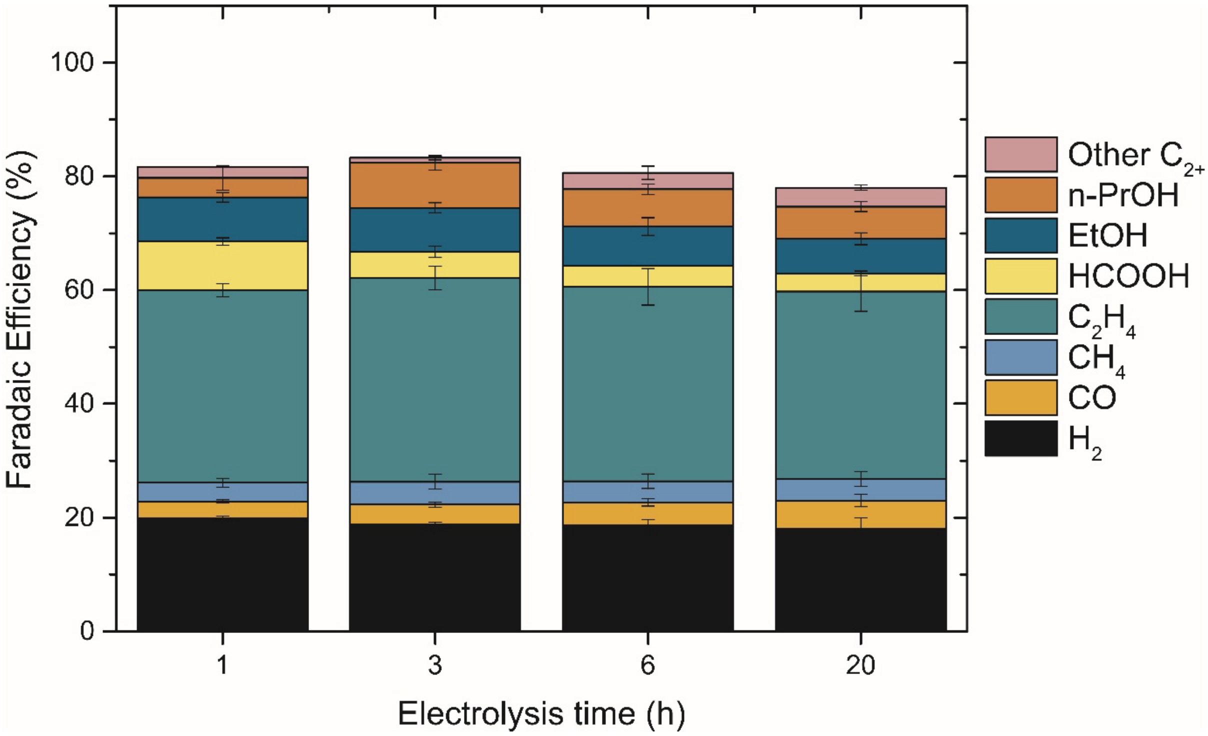

Figure S12c. Faradaic Efficiency of all products as function of electrolysis time for the Cu_2O electrode with Nafion ionomer. Other C_{2+} products include ethane, acetate, ethylene glycol, acetone, ethanal and allyl alcohol.

Cu_2O-Naf-Sus

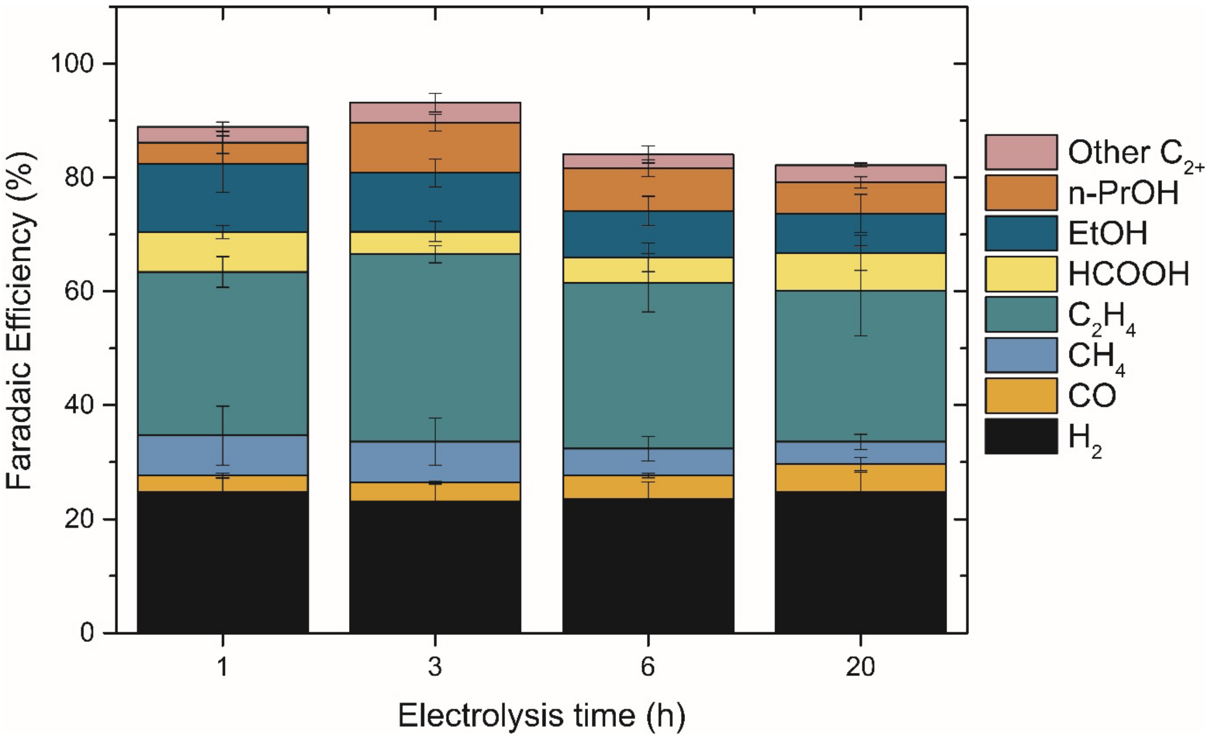

Figure S12d. Faradaic Efficiency of all products as function of electrolysis time for the Cu_2O electrode with first a Nafion and then a Sustainion ionomer layer. Other C_{2+} products include ethane, acetate, ethylene glycol, acetone, ethanal and allyl alcohol.

Cu_2O-Sus-Naf

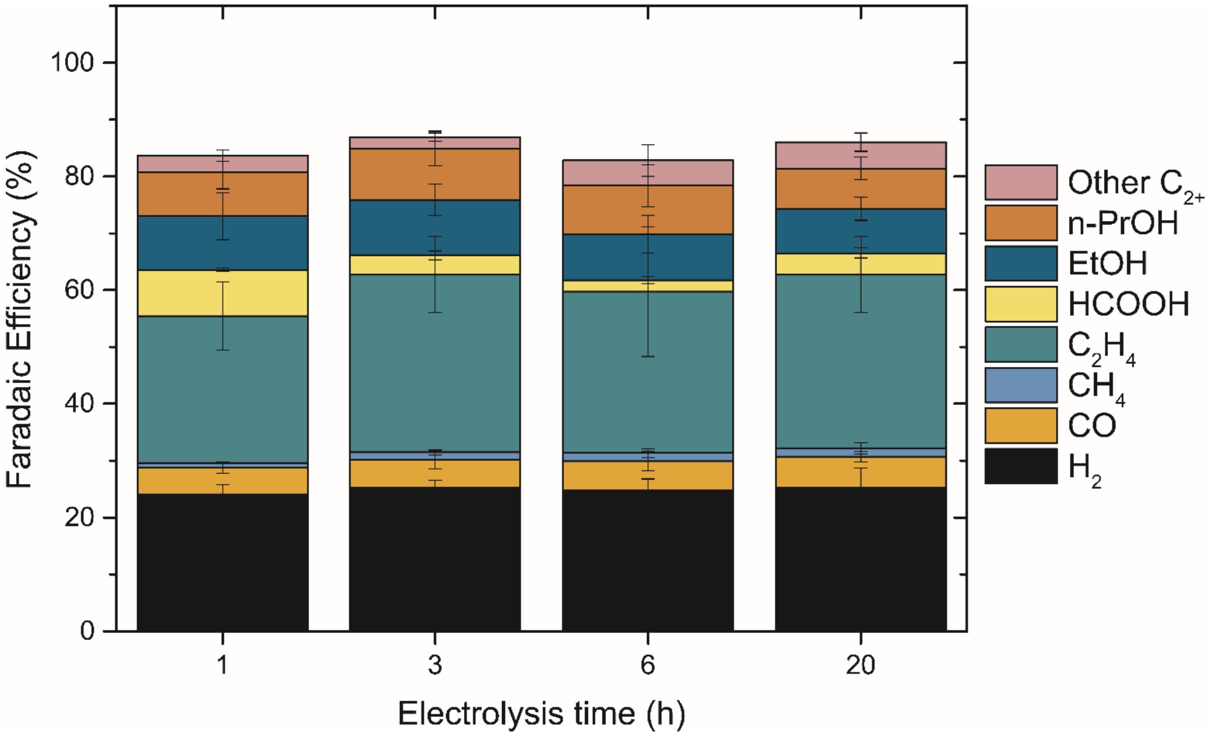

Figure S12e. Faradaic Efficiency of all products as function of electrolysis time for the Cu_2O electrode with first a Sustainion and then a Nafion ionomer layer. Other C_{2+} products include ethane, acetate, ethylene glycol, acetone, ethanal and allyl alcohol.

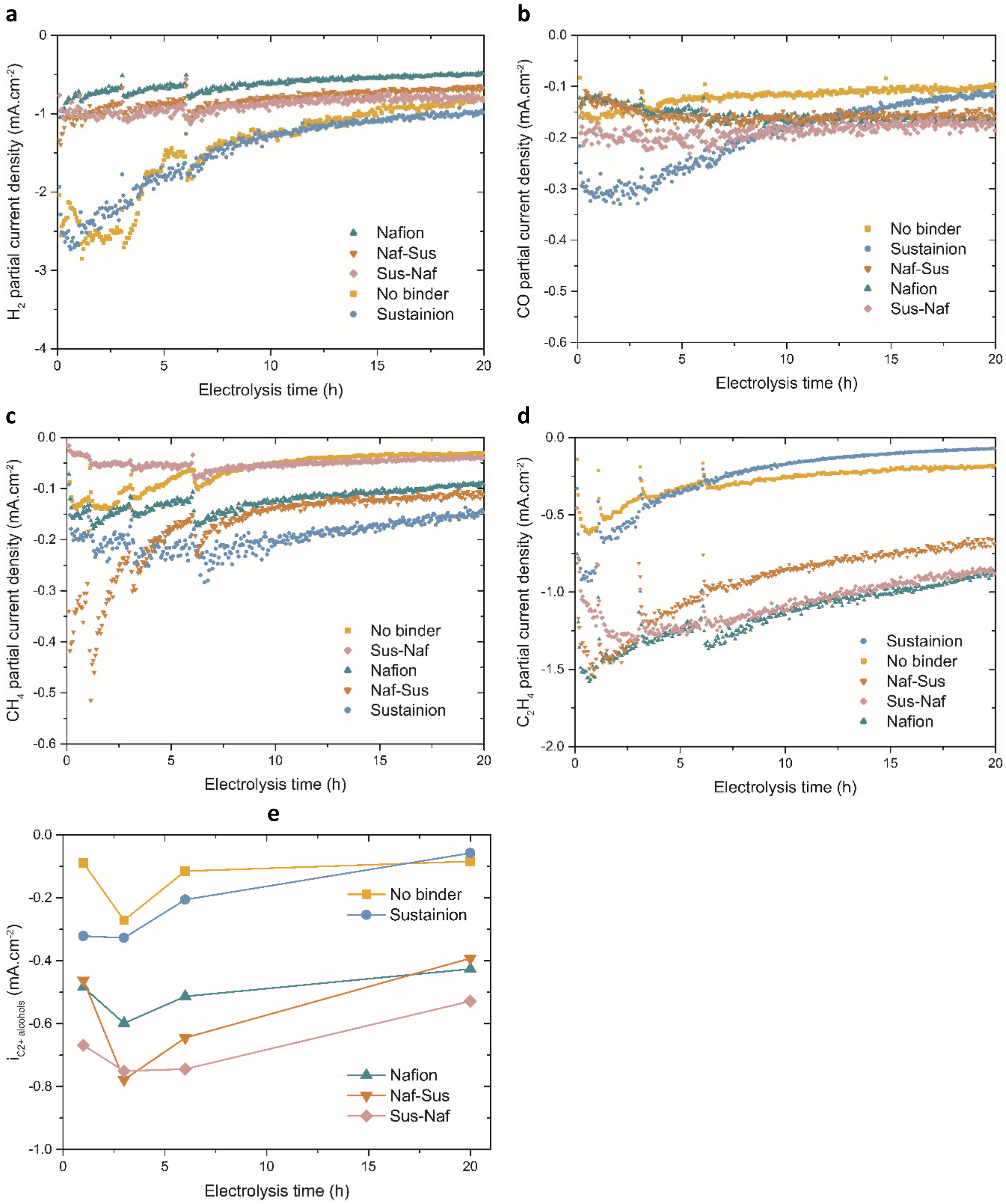

Figure S13. Partial current density to **a.** H_2, **b.** CO, **c.** CH_4, **d.** C_2H_4 and **e.** C_{2+} alcohols over time. C_{2+} alcohols include ethanol, n-propanol and allyl alcohol.

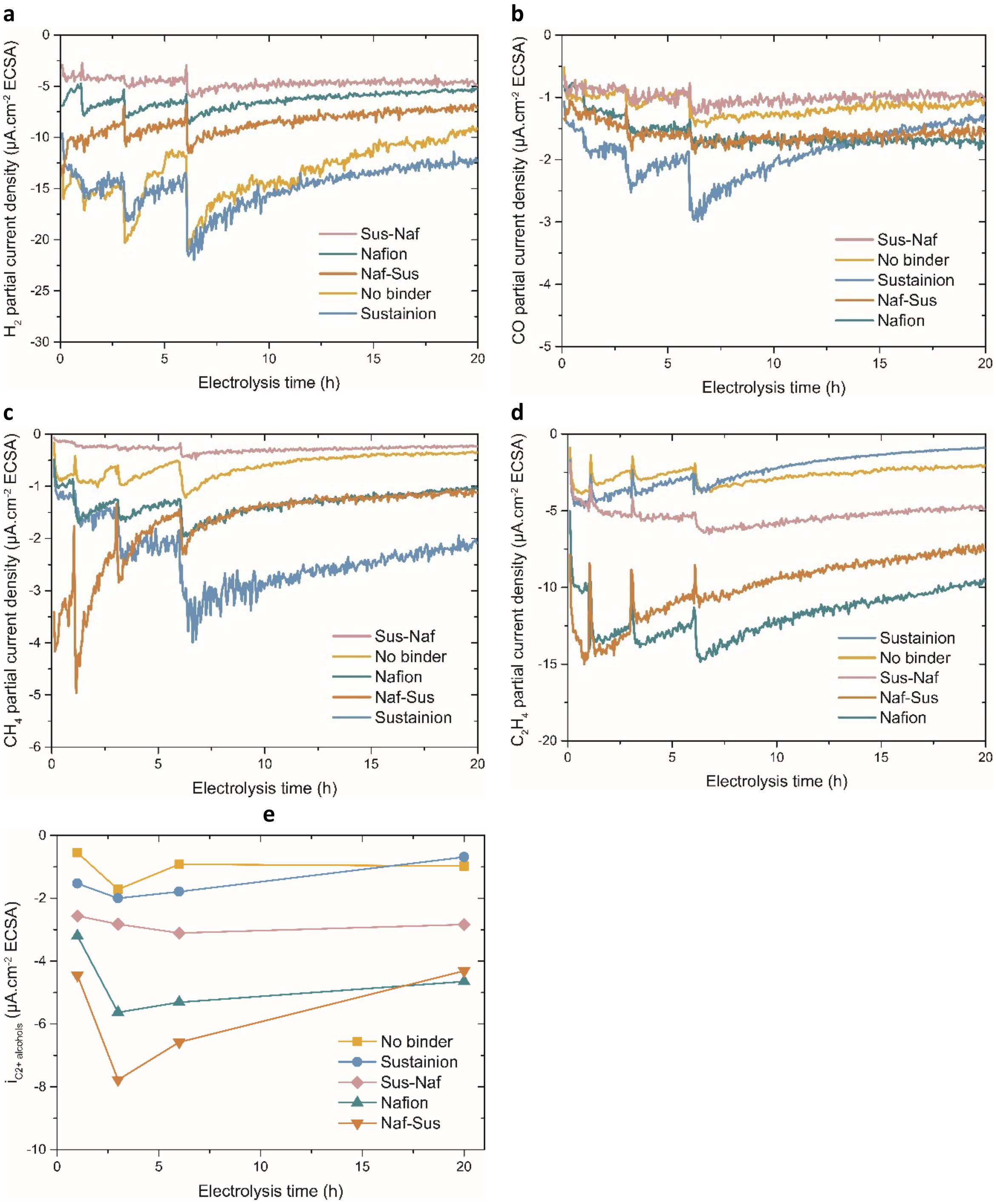

Figure S14. ECSA normalized partial current density as a function of electrolysis time to **a.** H_2, **b.** CO, **c.** CH_4, **d.** C_2H_4 and **e.** C_{2+} alcohols over time. C_{2+} alcohols include ethanol, n-propanol and allyl alcohol.

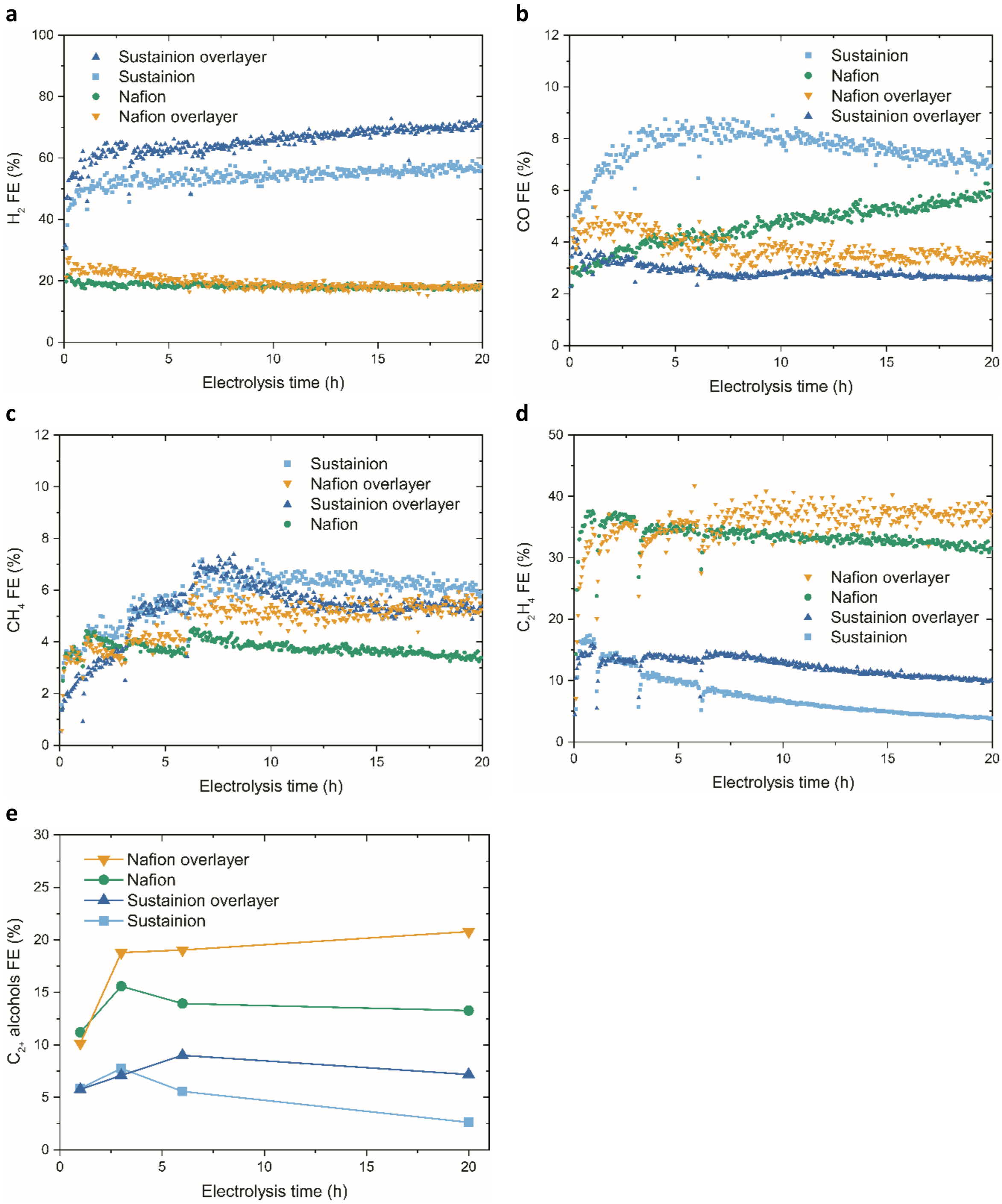

Figure S15. Faradaic Efficiency to **a.** H_2, **b.** CO, **c.** CH_4, **d.** C_2H_4 and **e.** C_{2+} alcohols over time for Sustainion and Nafion electrodes with the binder either added in the catalyst ink or separately as an overlayer. C_{2+} alcohols include ethanol, n-propanol and allyl alcohol.

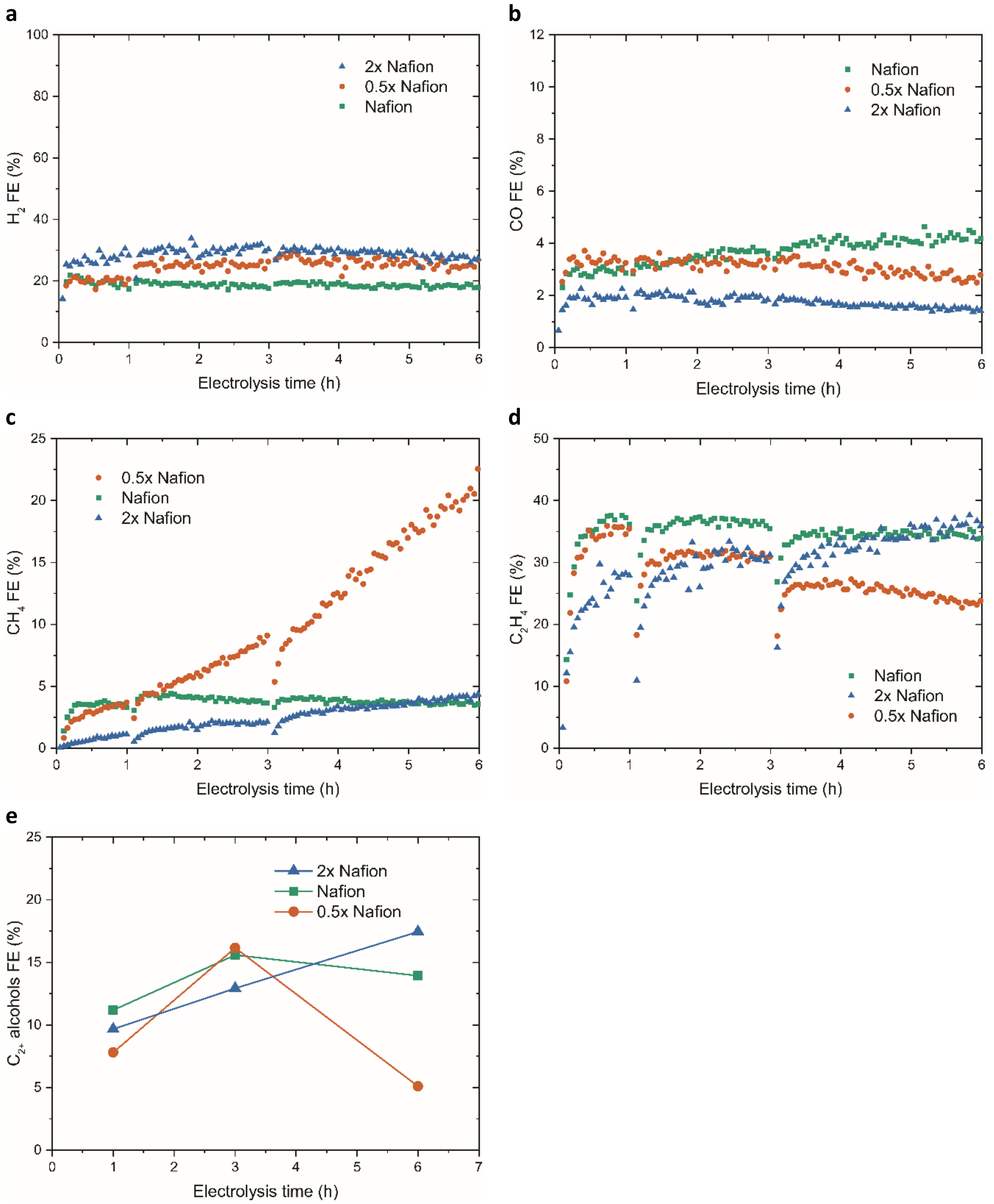

Figure S16. Faradaic Efficiency to **a.** H_2, **b.** CO, **c.** CH_4, **d.** C_2H_4 and **e.** C_{2+} alcohols during 6 hours electrolysis for three electrodes with different Nafion loading. C_{2+} alcohols include ethanol, n-propanol and allyl alcohol.

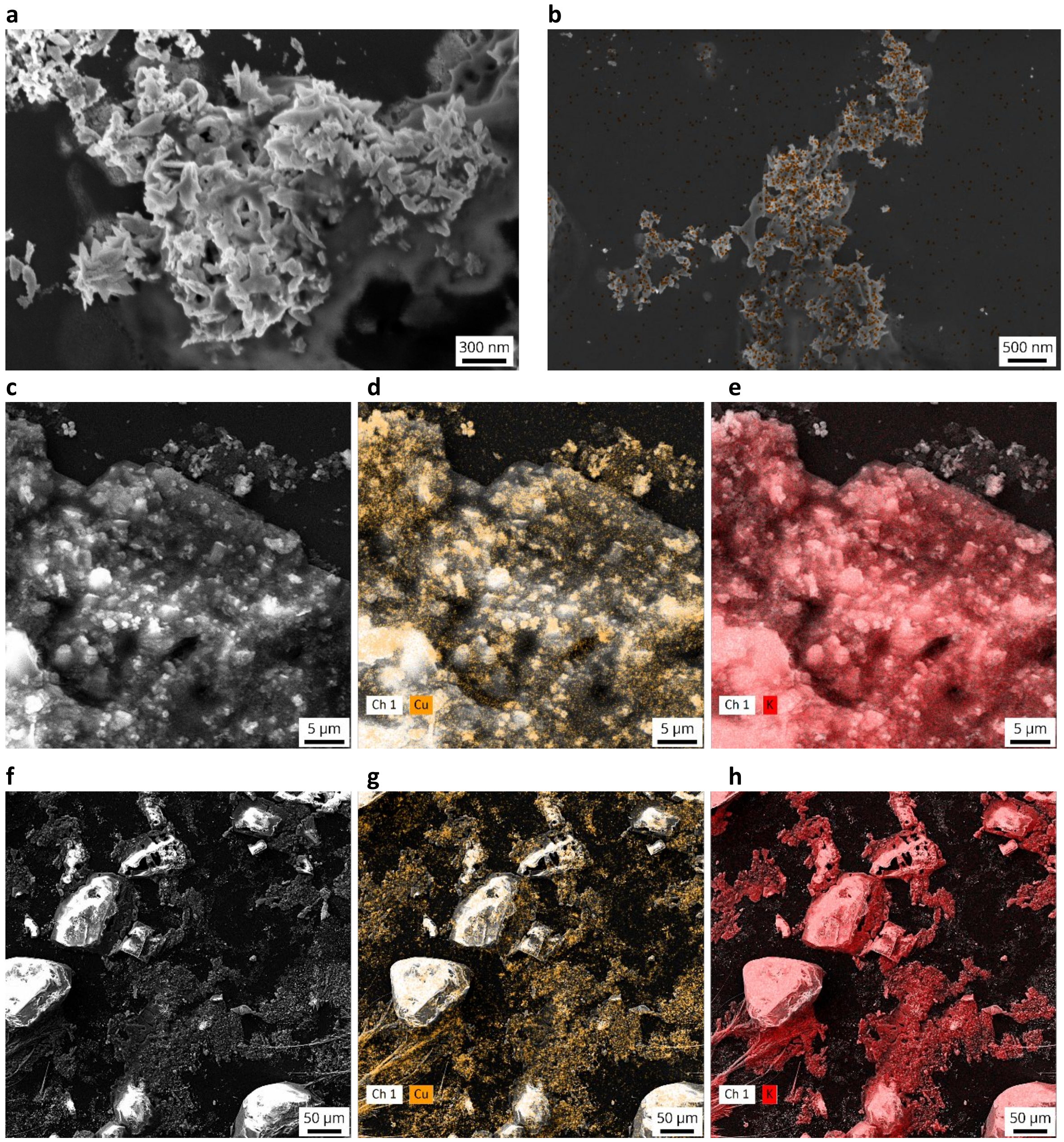

Figure S17. SEM-EDX images and maps at various magnifications of the electrode without ionomer binder after catalytic testing. Cu is colored orange. K is colored red, overlapping with the position of (bi)carbonate salt deposits.

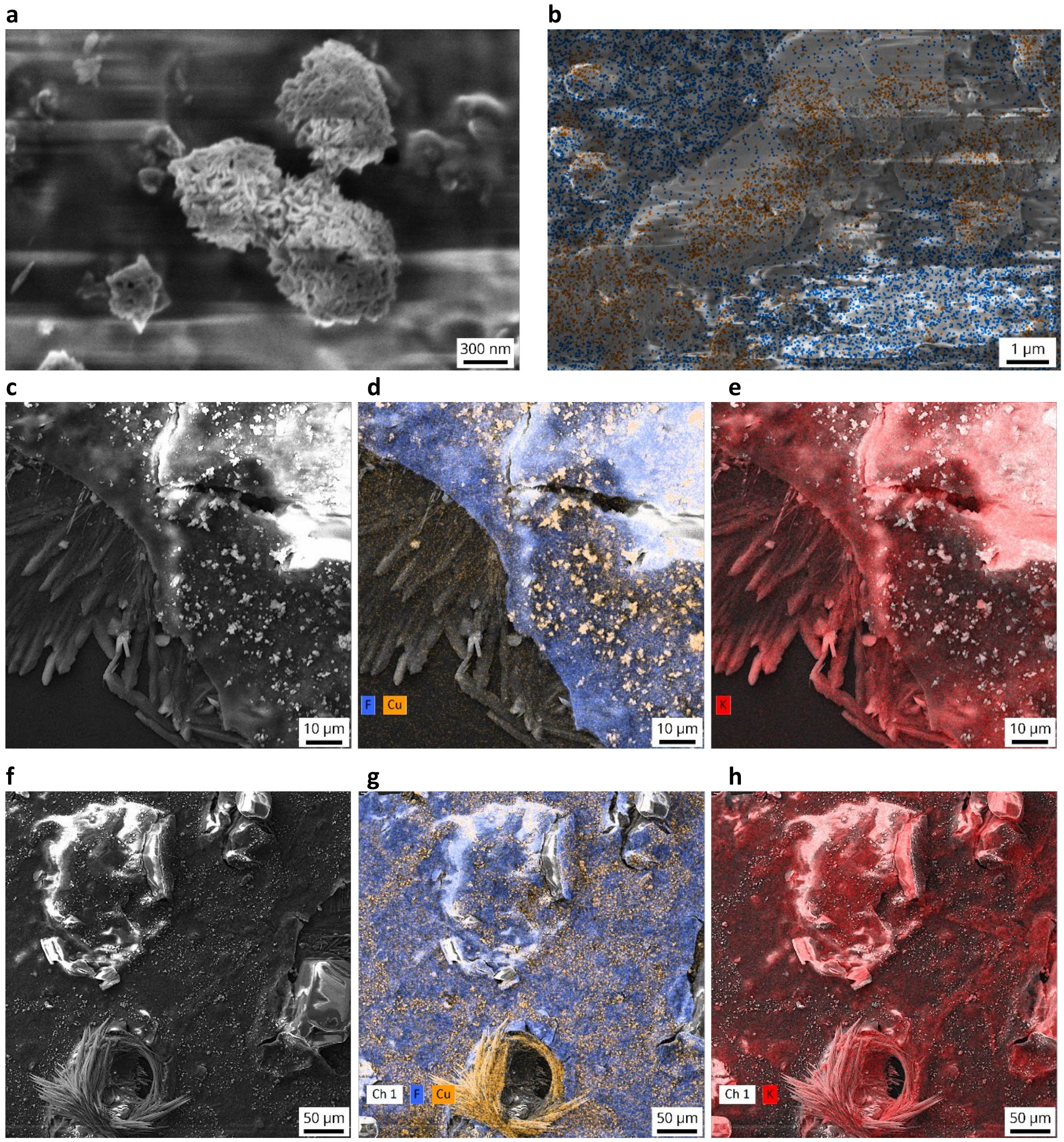

Figure S18. SEM-EDX images and maps at various magnifications of the electrode with Nafion ionomer binder after catalytic testing. Cu is colored orange. F is colored blue, originating from the PTFE backbone of the Nafion ionomer. K is colored red, overlapping with the position of (bi)carbonate salt deposits.

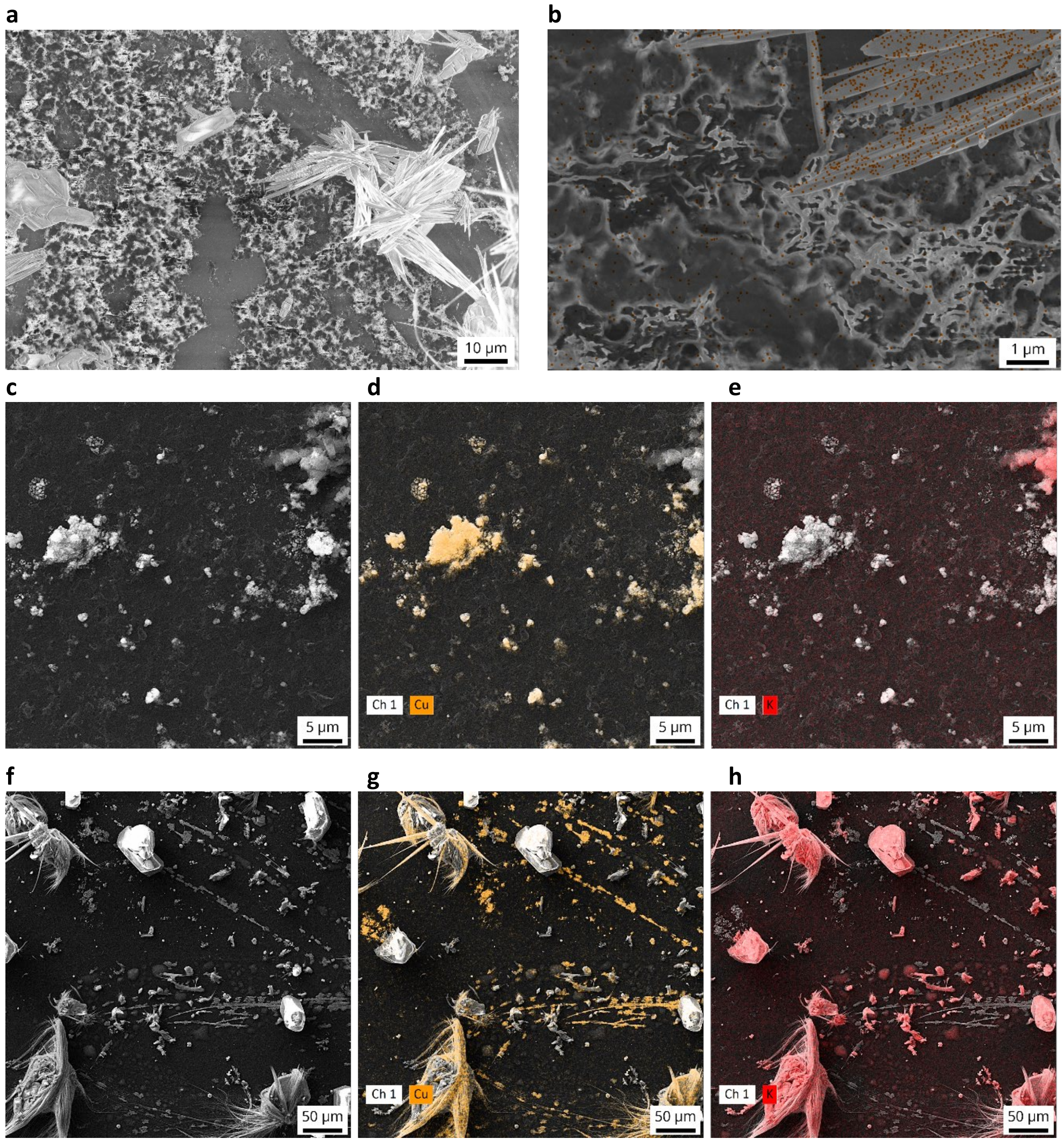

Figure S19. SEM-EDX images and maps at various magnifications of the electrode with Sustainion ionomer binder after catalytic testing. Cu is colored orange. K is colored red, overlapping with the position of (bi)carbonate salt deposits.

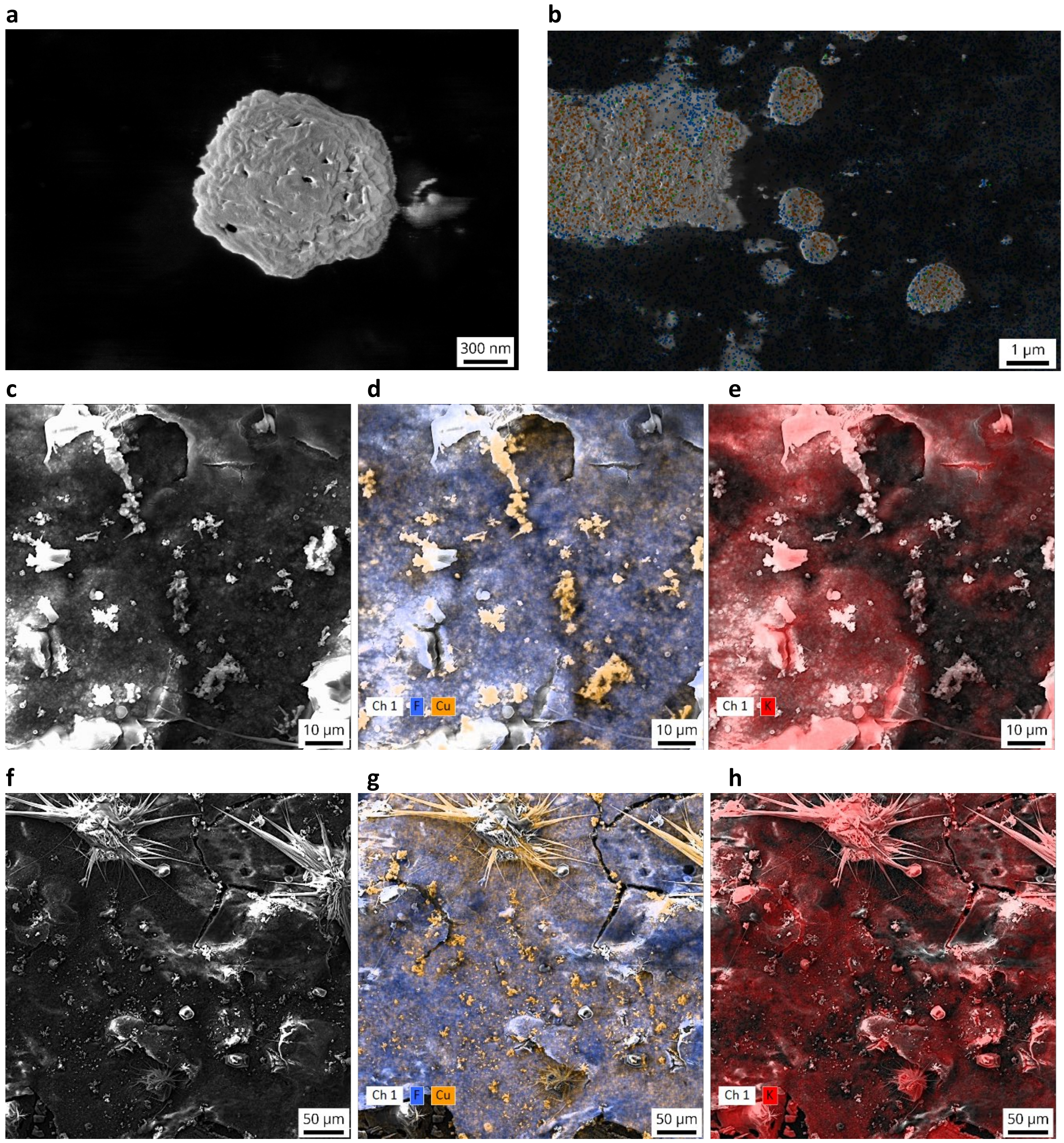

Figure S20. SEM-EDX images and maps at various magnifications of the electrode with Naf-Sus ionomer binders after catalytic testing. Cu is colored orange. F is colored blue, originating from the PTFE backbone of the Nafion ionomer. K is colored red, overlapping with the position of (bi)carbonate salt deposits.

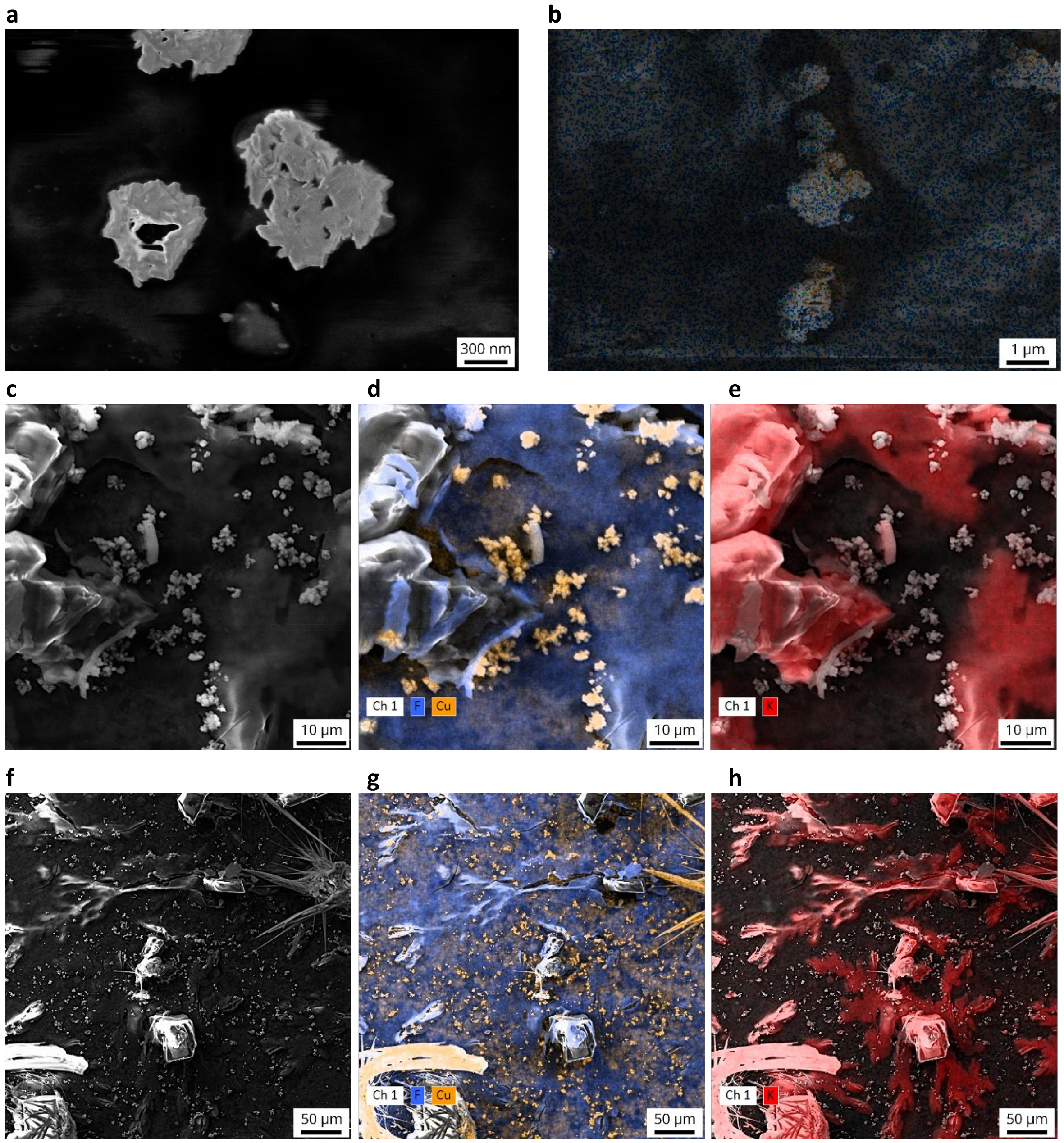

Figure S21. SEM-EDX images and maps at various magnifications of the electrode with Sus-Naf ionomer binders after catalytic testing. Cu is colored orange. F is colored blue, originating from the PTFE backbone of the Nafion ionomer. K is colored red, overlapping with the position of (bi)carbonate salt deposits.

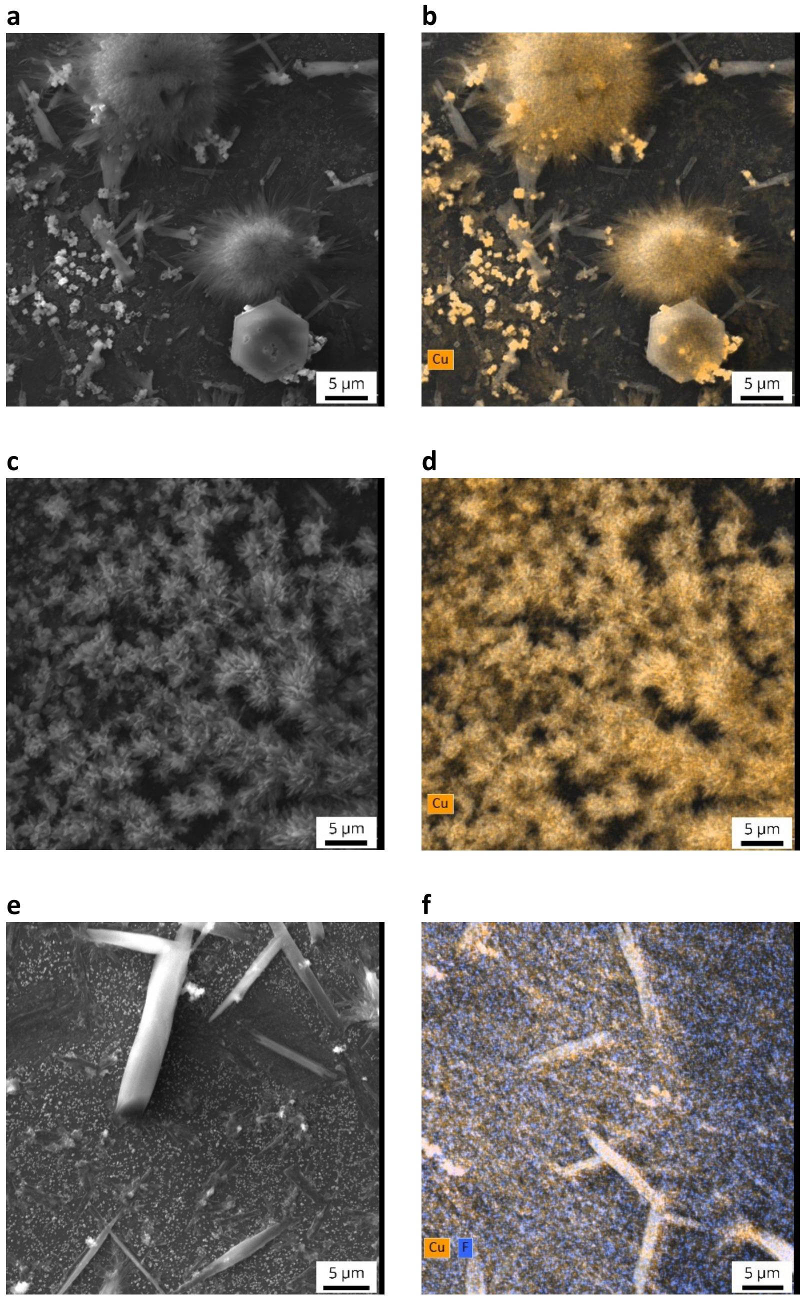

Figure S22. SEM images and Cu and F EDX maps of the electrodes **a-b.** without binder, **c-d.** with Sustainion and **e-f.** with Nafion after 15 minutes at -1.0 V vs RHE. Cu is colored orange, F is colored blue.

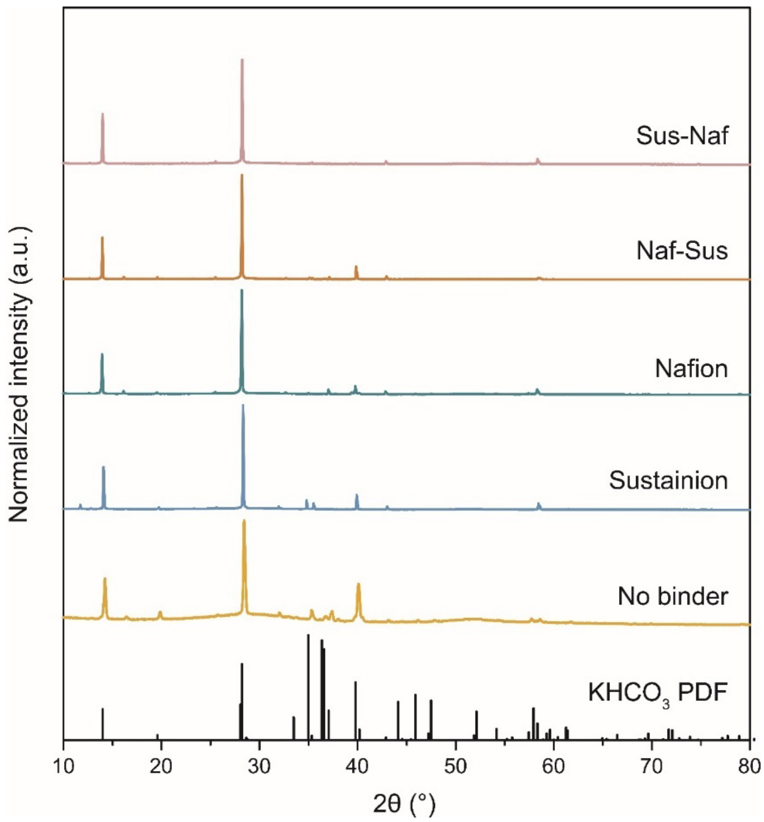

Figure S23. XRD patterns of electrodes after catalytic testing at -1.0 V for 20 hours. All patterns are normalized to the peak at 28°.

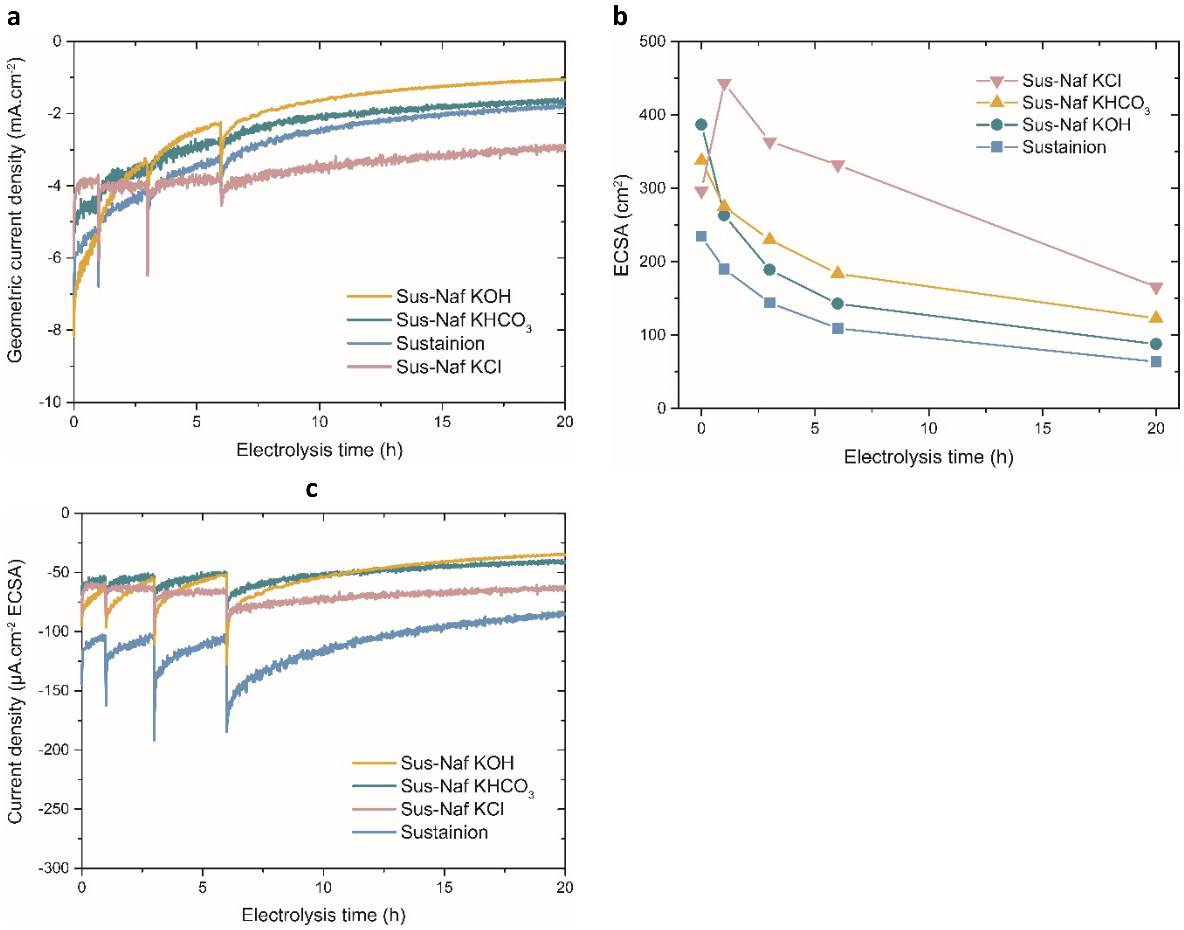

Figure S24. Activity data of 20 hour stability tests on the Cu_2O-based electrodes containing only Sustainion and containing a double layer of Sustainion and Nafion; ion-exchanged with KCl, KOH and $KHCO_3$. **a.** Geometric current density over time. **b.** Cu ECSA as determined from double layer capacitance measurements as function of time. **c.** ECSA normalized current density over time, obtained by normalizing for the average ECSA at the start and end of each time interval.

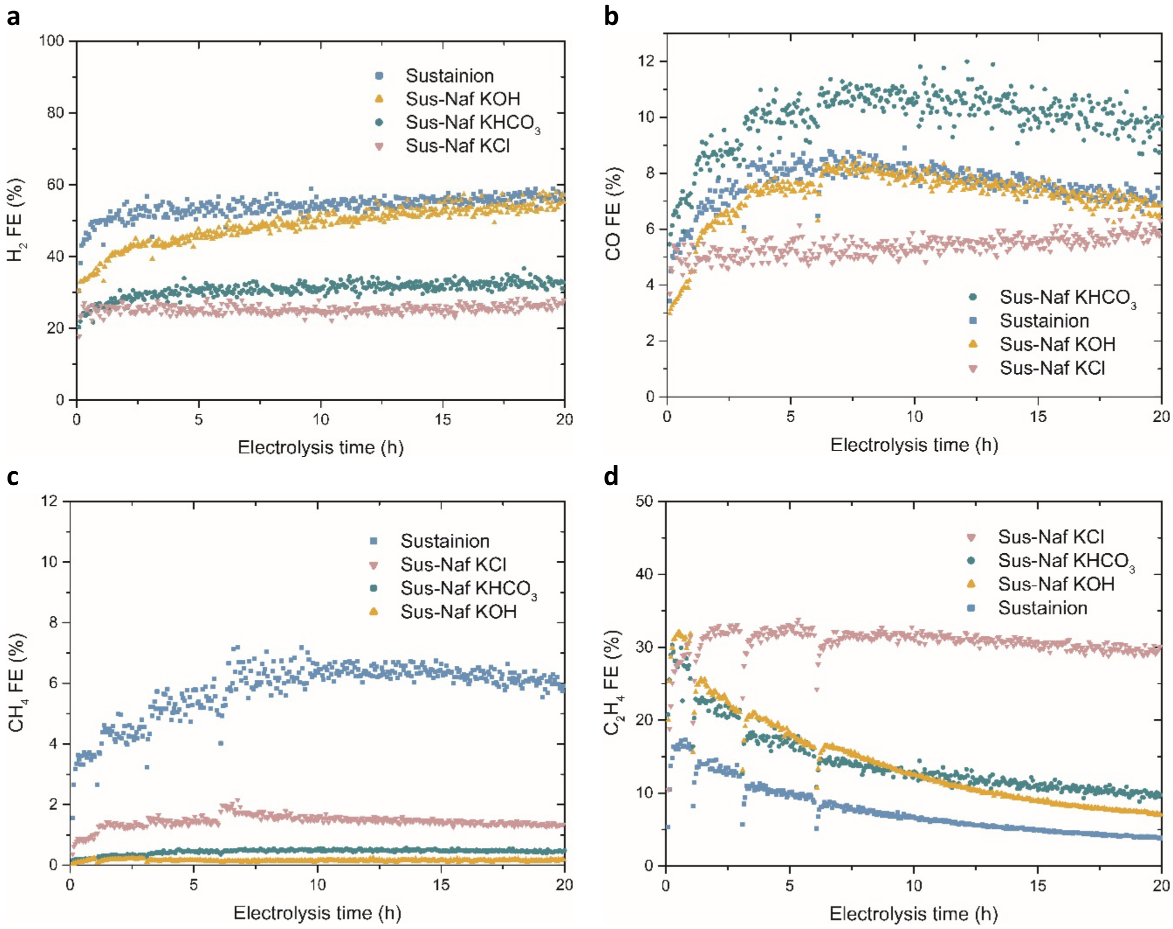

Figure S25. Results of 20 hour stability tests on the Cu_2O-based electrodes containing only Sustainion and containing a double layer of Sustainion and Nafion; ion-exchanged with KCl, KOH and $KHCO_3$. The **a.** H_2, **b.** CO, **c.** CH_4, and **d.** C_2H_4 FE are shown as function of time.

Cu_2O-Sus-Naf $KHCO_3$-exchanged

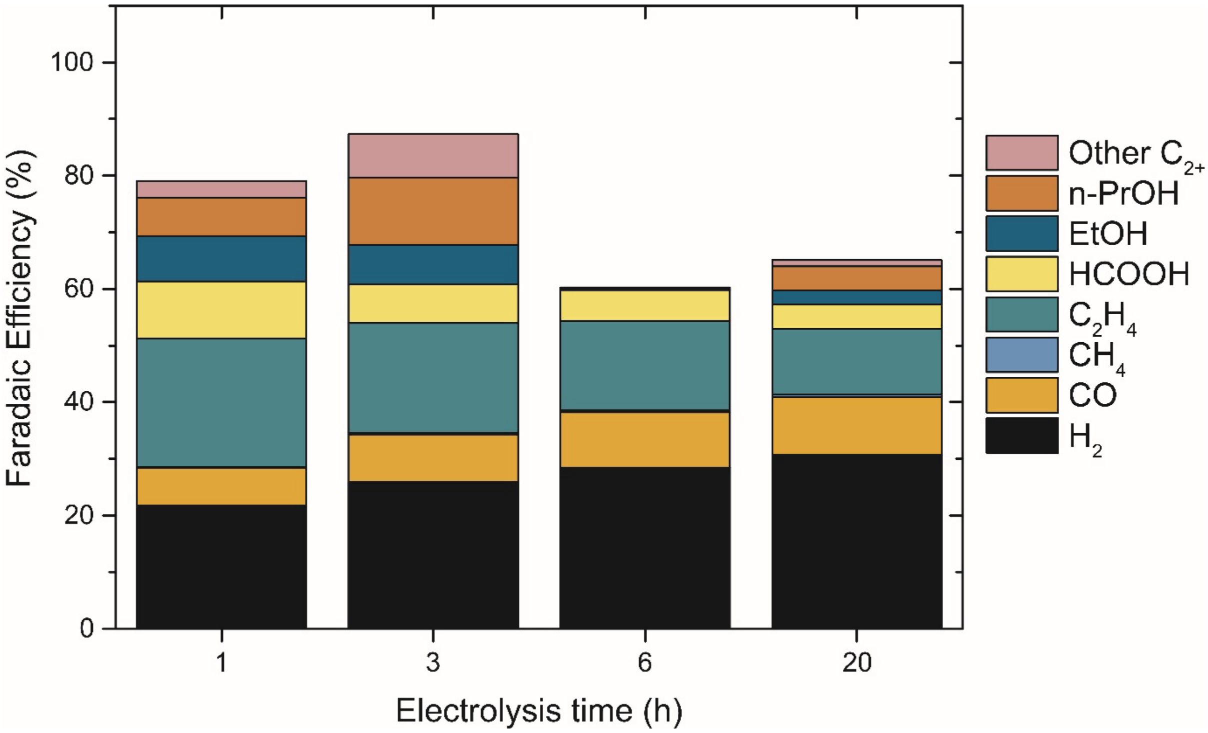

Figure S26. Faradaic Efficiency of all products as function of electrolysis time for the Cu_2O electrode with first a Sustainion and then a Nafion ionomer layer. The Sustainion layer was ion-exchanged in $KHCO_3$. 'Other C_{2+} products' include ethane, acetate, ethylene glycol, acetone, ethanal and allyl alcohol.

Cu_2O-Sus-Naf KOH-exchanged

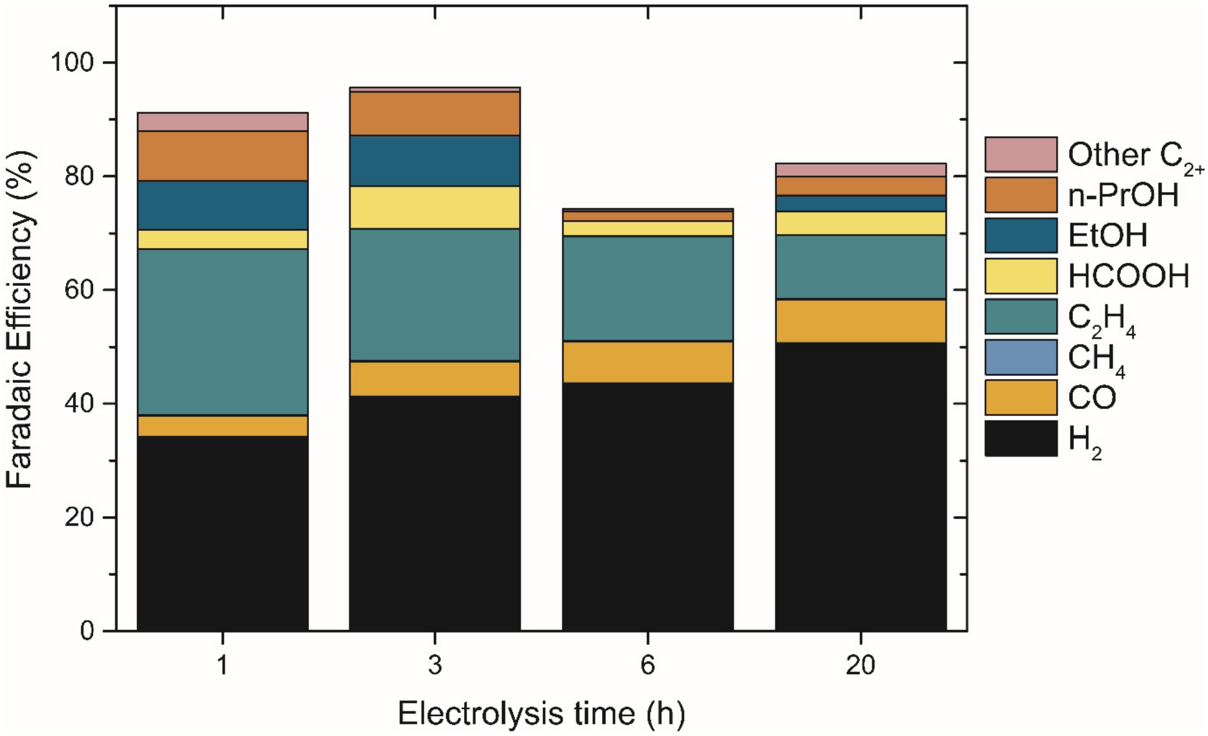

Figure S27. Faradaic Efficiency of all products as function of electrolysis time for the Cu_2O electrode with first a Sustainion and then a Nafion ionomer layer. The Sustainion layer was ion-exchanged in KOH. 'Other C_{2+} products' include ethane, acetate, ethylene glycol, acetone, ethanal and allyl alcohol.
